# Perceptions of the oral health of their patients reported by Brazilian medical residents in psychiatry

**DOI:** 10.1371/journal.pone.0282945

**Published:** 2023-04-17

**Authors:** Luciane Miranda Guerra, Brunna Verna Castro Gondinho, Rodrigo Almeida Bastos, Felipe dos Santos Silva, Júlia Vitório Octaviani, Jasmine de Matos Cavalcante, Egberto Ribeiro Turato

**Affiliations:** 1 Department of Health Sciences and Children’s Dentistry, Piracicaba Dental School, Campinas State University (FOP/UNICAMP), Piracicaba, São Paulo, Brazil; 2 Faculty of Dentistry and Nursing of the State University of Piauí (UESPI), Parnaíba, Piauí, Brazil; 3 Department of Nursing, Federal University of Paraná (UFPR), Curitiba, Paraná, Brazil; 4 Department of Medical Psychology and Psychiatry, School of Medical Sciences - State University of Campinas (FCM/ UNICAMP), Campinas, São Paulo, Brazil; 5 Graduation in Medicine at the University of Campinas (UNICAMP), Campinas, São Paulo, Brazil; 6 Department of Psychiatry, School of Medical Sciences, State University of Campinas (FCM/ UNICAMP), Campinas, São Paulo, Brazil; Shahid Beheshti University of Medical Sciences, School of Dentistry, ISLAMIC REPUBLIC OF IRAN

## Abstract

Persons with severe mental disorders have higher chances of presenting oral health problems than persons in the general population. Mental disorders are considered *public-health problems* worldwide. Interpreting statements on oral health made by medical students receiving instruction in psychiatry is essential to provide elements for reflection on their difficulties, barriers or limited knowledge in managing their patients’ oral health. Based on the presupposition that doctors provide their patients with no instruction or support concerning oral complaints, because these do not symbolize the same challenges as those emerging from severe mental disorders. The aim of this study was to interpret symbolic meanings of statements expressed by medical residents in psychiatry at a specialized university outpatient clinic, about their patients’ oral health. Qualitative research using the Qualitative-Clinical method was conducted, adopting the theoretical reference of medical psychology. Data were analyzed by the Clinical-Qualitative Content method and the Seven-Step technique. Based on 06 interviews analyzed, the following categories emerged: “What do I do? *Psychiatrists’ dilemmas about not understanding their role in patients’ oral health;* and *“Dentists do not participate in Balint’ so-called “collusion in anonymity”*. It was concluded that among others, the paradigm consisted of a sociological composition, therefore it was stronger than personal decisions that might eventually oppose the barriers to interdisciplinary work posed by the institutional model, which were intertwined with paradigmatic barriers. Thus, specialist training should include a dental perspective, so that oral examinations would always be included in the physical examinations, from an interdisciplinary perspective of the integrity of health-care.

## Introduction

The oral health of patients with mental disorders is critical since certain diseases may cause or worsen other diseases or conditions that are significantly associated with mental disturbances. Periodontitis, for example, is a risk factor for cardiovascular disease [1] and for poor glycemic control [2]. Cardiovascular diseases are associated with mental disorders. The pain and discomfort associated with oral diseases may result in dietary difficulties that lead to different levels of malnutrition. Poor oral health may affect the quality of daily life, well-being and self-esteem of these patients [3]. Considering that in general, patients with mental disturbances are predisposed to manifesting effects on their self-esteem, consequently, oral problems may have an even greater impact on them [3].

Studies have suggested that persons with severe mental diseases have a significantly higher chance of presenting oral health problems than those in the general population [1, 4]. This is due to various reasons, such persons with mental disturbances being incapable of perceiving their oral health problems, feeling dental phobia, having difficulty with gaining access to health services, experiencing side effects of psychiatric medications. Whereas dentists may lack knowledge and have inadequate attitudes towards these patients, such as “not attending” them because they are afraid of suffering aggressions, perform acts of omission in the face of acute conditions because they lack pharmacological knowledge about the medications being used by patients, particularly due to prejudice and fear of the unknown, etc. Frequently, these patients find dental treatment to be a challenging experience, due to a certain apathy or limited cooperation—characteristic of some disorders -, a low level of adaptability to new dental prostheses, fear and other distressing emotions [5]. Despite the health professionals’ increase in focus on the physical health of persons with severe mental diseases, this does not appear to be accompanied to the same extent by an increase in focus on the oral health of these patients [6]. People with severe mental illness are 2.7 times more likely to lose all their teeth in comparison with the general population [6].

Bearing in mind that mental disturbances comprise a wide range of problems and that their prevalence worldwide characterizes them as *public health problems* [7], and that in Brazil 30% of the adults have symptoms of common mental disturbances [8], the pertinence of this study is clear. This concerns a growing demand in a population that is predisposed to greater risk of having oral problems not only because of being affected by various symptomatic psychiatric expressions but because they may develop new disorders or aggravation of previously existing conditions.

Whereas the biopsychosocial concept, an approach that has been recovered in the medical and scientific fields, allows us to understand the facets of which the human [9] being is composed. The constituents of biological and physiological perspectives are limited for the purpose of contemplating the meaning of phenomena of an emotional order of the subject. This demands the need for a humanistic perspective that integrates, includes and seeks to investigate the statements of mental phenomena.

It is fundamental to understand the meanings attributed to oral health by the medical students—residents receiving instruction in psychiatry—for the purpose of interpreting the statements they made about this question. Thus, provide elements for reflection on the difficulties, barriers or limitations of knowledge that psychiatrists might have in dealing with the oral health of their patients.

We began with the empirical presupposition that these professionals do not instruct their patients about care of the oral cavity or provide them with any support concerning their oral complaints, since these are not symbolic of the same challenges that emerge in the clinical setting as those that are related to the severity of mental disorders. The aim of this study was to interpret the symbolic meanings that medical residents of psychiatry. at a specialized outpatient clinic of the university, expressed in their statements about the oral health of their patients.

## Materials and methods

A humanistic study was conducted with the use of the Clinical qualitative research method [10], which would allow understanding of the meanings that the doctors of psychiatry, residents of a public university in the interior of Brazil, attributed in a report on the oral health of their patients. The theoretical reference adopted for analysis and discussion of the findings of this study was that of medical psychology. As this was a Balintian type of data analysis, the analysis was performed from the psychodynamic perspective, which gives meaning to the speech.

Construction of this research project counted on the participation of Members of the Laboratory of Clinical- Qualitative Research (LPCQ) of the State University of Campinas, made up of a multidisciplinary team composed of doctors, nurses, psychologists, nutritionists and dentists with experience in conducting interviews not directed towards seeking nuclei of meanings in floating readings of the transcribed content.

We emphasize that in Brazil, Dental education begins with the undergraduate course in Dentistry and in a different form in relation to medical education; that is to say in a manner independent of medical studies. They are distinct courses. They are therefore, two different types of educational systems with paradigmatic and technical inter-relations.

This manuscript was constructed in accordance with the criteria of the COREQ checklist (Consolidated criteria for reporting qualitative research).

### Study location

The study was conducted at the Specialized Psychiatric Outpatient Clinic of the General Hospital of UNICAMP, scenario of practice of these medical residents, which is intended for the education of specialists in psychiatry. The research was conducted in this clinic because it is in this place where the patients with severe disorders are followed up longitudinally. It is, therefore, not an emergency service. It is a service the provides continuity of care. Thus, there is a bond between the doctors and their respective patients. This is necessary to enable investigation into what the oral health of patients means to these doctors. Moreover, these patients are not in a state of urgency (in a crisis or flare-up), which allows the doctor to attend to different needs of the patient, in addition to the specific mental problem. In the conventional hospital environment, this would not be possible, since the service provided is of an urgent nature, making it impossible to follow up the cases and provide longitudinal care. This outpatient clinic (HC UNICAMP) was specifically chosen for convenience, as the researcher is a professor at the same University and is interested in recognizing students’ potential learning needs for the qualification of patients’ health care.

The Outpatient clinic is integrated into a Hospital School at university level of high complexity, where all the medical specialties work. This outpatient clinic attends to adults with severe mental disorders (schizophrenia, bipolar disorder, refractory depression, severe phobias etc.), psychiatric patients who have been discharged from the Hospital wards, and severe cases that have been evaluated by the Urgency and Emergency service (all of whom are referred from the public health network.) Each resident attends an average of two patients per hour. The residents generally attend the patient alone, but they are supervised *in loco* by professors of the Department of Psychiatry. Meetings for discussing cases are held on a weekly basis The State University of Campinas (municipality that is located in a metropolitan region in the interior of the State of São Paulo and, therefore, serves over 2 million people from all over this region) has over 60 undergraduate courses, among them Dentistry and Medicine. The Campus pertaining to each of the latter courses is, however, located in two different cities.

### Sampling and recruiting

The universe of this study consisted of 18 resident doctors, who could potentially have been selected for the interviews. The sample was constructed in an *intentional* and *sequential* manner, and the inclusion criteria were: the participant had to be a medical resident of the Psychiatric Outpatient Clinic service of the Hospital School of UNICAMP, regularly enrolled; must be inadequate physical, emotional and intellectual conditions at the time of data collection so that there would be no harm done to the attribute of methodological validity, such as that expected in the obtainment of information verbalized in interviews.

Before beginning with the interviews, the researcher, a woman dentist, PhD in Dentistry, first author of the article, and responsible for the research, who conducted all interviews, had previous experience in holding in depth interviews, completed the stage of acculturation in the clinical setting, where the research would take place. The researcher frequented the Outpatient Clinic for two months, observing the routine of the work, and making notes in the field. Moreover, she participated in meetings of the Residents’ Group, discussion of cases between residents and professors, frequented the premises of the outpatient clinic and talked with the local nursing staff. The field notes allowed registration of the context observed, and afterwards were subsidiaries in the Clinical-Qualitative Content Analysis for understanding the experiences reported by the interviewees. In this period, ‘understood as being *the establishment of relations with the habitual persons in the environment (health teams*, *bureaucrats*, *patients)*, enabled the initial contact between the researcher and the subject/environment. This favored the inversion and understanding of the researcher, about the way the universe investigated functioned [11]. Initially, the interviewees were approached face-to-face, and they were invited to participate in the research. As from March 2020, the contacts were made by email. The date and time for the interview were previously scheduled according to the preference and convenience of the interviewees;

The sample was closed according to the criterion of theoretical saturation of information, which included new participants until such time as the researcher perceived that no new data was being added during the interviews. The interviews began to show patterns of repetition [12, 13].

### Data collection

The instruments used for data collection were the semi-directed interview of in depth open questions, complemented with the field notes.

The interviewing technique used was by opening with a trigger question: “*What does taking care of the oral health of patients mean to you*. *as a medical resident*?*”* During the course of the interview, in depth questions were used to expand and elucidate the statements.

Data were collected in the period between November 2019 and December 2020. At the beginning of the research the researcher was present in the outpatient clinic, presented her research to all present at the team meeting, and afterwards scheduled meetings with the doctors at appropriate times and days. Before each of the interviews began, the interviewer introduced to the interviewee and read the free and informed consent request (FICR) in full. At the conclusion of the reading, he asked the interviewee if he/she had any questions and if he/she agreed to participate. After this, the interviewee signed two copies of the FICR and the interview began. The interviews had no predetermined time limit. Each interviewee participated in the research only one time. These interviews were afterwards audio recorded and transcribed in full.

Due to the pandemic, the interviews were held in two distinct ways: the first four interviews were held face-to-face at the outpatient clinic after the interviewer had read out the Term of Free and Informed Consent (FICR) to each interviewee. As from March 2020, due to social isolation imposed by the sanitary authorities, the contacts were made by email. A first email was then sent to the resident doctors selected, explaining the purpose of the research and inviting them to an individual online interview through the Google Meet platform. In this email there was also a link so that the doctors could access the FICR online, if they accepted the invitation.

After virtual acceptance of the FICR, the other two interviews were scheduled and held virtually by means of the Google Meet Platform. On conclusion of the period of social isolation, the researcher went to the clinic with printed copies of FICRs and collected the interviewees’ signatures on these FICRs.

All the interviews were audio recorded with the consent of the interviewees. The data were transcribed in full. There was no need to repeat any interview

### Ethical approval

The research project was approved by the Ethics Committee of the School of Medical Sciences of the State University of Campinas, SP (CAAE: 20229219.2. 0000.5404) and the number of this process was 3.624.220. All the procedures were in accordance with the ethical standards. During the course of the research, in July 2020, as a result of social isolation imposed by COVID 19, an amendment was made to the Ethics Committee/CEP, requesting authorization to hold the interviews by remote means (online), which was approved.

### Data analysis

**Fig 1:**
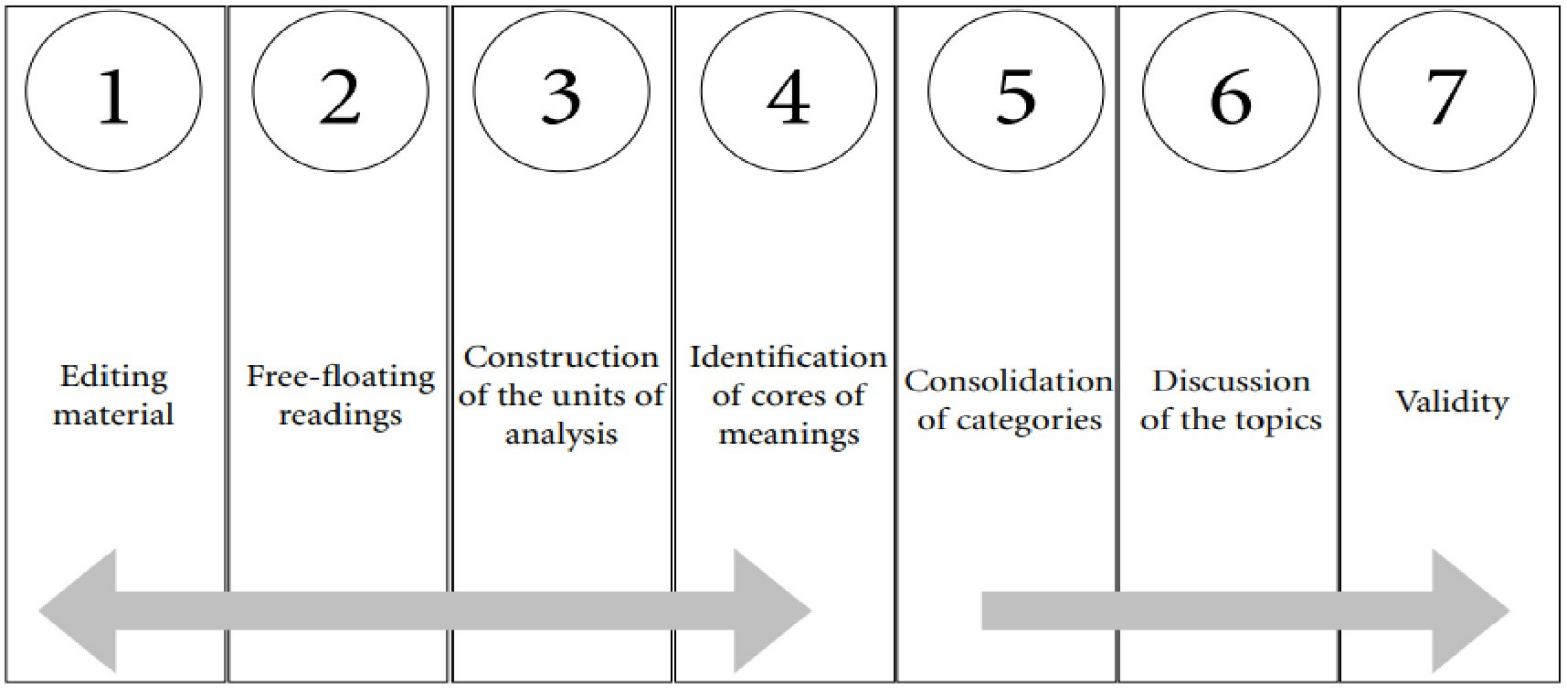
Flow chart of the Clinical-Qualitative Content Analysis.

Data were analyzed by means of the Clinical-Qualitative Content method. The *corpus* is the set of interviews transcribed in full and the complementary notes taken in the field. In the technique entitled “Seven Steps” of Faria-Schützer [14], the material transcribed in full is read in the floating manner and re-read when units of analysis (codes) emerge. After this they were consolidated into categories of discussion that were validated by the peers at meetings of the research group to which the authors are affiliated. This analysis is demonstrated according to the flow diagram below (Fig 1).

All the co authors participated in all stages of the project as from Step 2. The data were validated together with the peer researchers of whom the LPC is composed.

No software was used for managing the data and no feedback was requested from the participants.

## Results

In total seven (07) medical residents were interviewed. All those invited to participate in the research accepted. However, one of the interviews was lost. The total number of valid interviews was therefore, 06 (six). In the interview, only the interviewer and interviewee participated. None of the students that were invited refused to participate in the study.

The psychiatry residents were invited to speak freely on whatever they felt about their experiences in relation to the oral health of their patients. The interviews had a duration of 20 to 30 minutes, according to the disposition of each interviewee in speaking about the subject. No software was used for analysis of the data. Two Categories emerged from the data analyzed; “What do I do? *Dilemmas of psychiatrists about not understanding the role they play in oral health of the patients;* and *“The dentist does not participate in the so-called “collusion in the anonymity” of Balint”*.

To protect the participants’ identity, each participant was designated with a letter “E” and a number corresponding to him. The demographic characteristics of the sample studied are described in Table 1.

**Table 1:** Characterization of respondents according to age, sex and period of medical residency.

Interviewee	Age	Sex	Period of medical residence
Interviewee 1 (I1)	27	Male	2^nd^ year (R2)
Interviewee 2 (I2)	33	Male	3^nd^ year (R3)
Interviewee 3 (I3)	29	Male	2^nd^ year (R2)
Interviewee 4 (I4)	30	Male	2^nd^ year (R2)
Interviewee 5 (I5)	33	Female	4^nd^ year (R4)
Interviewee 6 (I6)	25	Female	1^nd^ ano (R1)
Interviewee 7 (I7)	27	Female	2^nd^ ano (R2)

***“*** ***What do I do*?*” Dilemmas of the psychiatrist about not understanding his***/***her role in the patient’s oral health***

The lack of understanding about the specialties and the different disciplines in the field of health, supposedly caused prejudice.

**I1-** *“And*, *here what do I do*?*” The patient hardly performs tooth brushing*, *the patient is in pain*, *the patient’s dental appearance is bad*, *does the patient have halitosis or not*, *in the end*, *I don’t know”*.

This gap in knowledge is unfavorable to multidisciplinary relations, collaborating with the concentration of responsibilities in the hands of the physician, and consolidating practices that demonstrate that the concerns occur depending on the interface of the area in which the physician works. The areas of interface of the patient with severe mental disturbance are, for example, abusive use of substances, eating disorders, severe pathological behaviors with manifestations in the mouth. In the mentioned “border diseases” the psychiatrist acts. However, the factor that makes the doctor psychiatrist’s focus of attention grow remote, makes it complex for him to act.

**I1** “*Therefore*,*it is extremely common*, *for example*, *for me to refer a patient with schizophrenia to the gynecologist for the first time…She has schizophrenia*, *has never been to the gynecologist*, *because the problem has always been schizophrenia*, *but it is an entirely other dimension that needs to be seen and everything else*. *Whereas for the dentist to admit that he/she was even thinking about this*, *does not happen so frequently*.

The teaching of clinical psychiatry follows the logic of the biomedical paradigm, directed towards a clinical practice, not favoring interdisciplinarity, for example, with the dentist participating in discussions. Clinical practices obey a logic of construction of the paradigmatic areas, according to the reasoning of the epistemological models.

**I1***“Because I end up not knowing*, *is it perhaps because in undergraduate studies there is extremely little—almost nothing about oral health*? *I think I may have had one or two classes—maybe”*

***The dentist does not participate in the so*** ***called Balintian “collusion of anonymity”***

Each of the different areas of health have their community, their specific group, the sharing of clinical cases occurs within these groups, that is to say, the movements tend to occur within each nucleus of knowledge.

**I1** *“And (I have referred) another case of a patient of over 50 years of age and (who had) some complaints for tracing cancer”*.

“…I have also made some referrals in this sense (gynecologist). In search of symptoms, these things, knowing that it was a patient who was less active in the network, less demanding of the rights, and I ended up doing this sometimes.”

In the above segment, in which the speaker reports the ease of referring a patient for oncological tracing, we see that the specialist has made a speech that leads us to understanding that he does not include the dentist in this network. This does not mean that the doctor does not think it important to include the dentist, and it does not mean corporatism. However, it means that institutional functioning follows a logic of sharing tasks among those who are of the same professional area.

The dentist does not participate in this “collusion’’, because he/she does not form part of the group of doctors since he is not from the same area of university education. This does not mean that the doctor is negligent. But he sees these manifestations when they are related to the medical problem. The sociological organization between paradigms becomes determinant.

**I3** *“And we end up not knowing much about how to indicate or what to do to speak about this (about oral health)*.”

The personal clinical practice within the consulting room follows the logic of “micro” understanding about health, the support for complaints of a "non-health” [11]. The referrals among medical specialties occur, but limit communication with non-medical areas.

**I1** *“I keep on thinking that the ease I have*, *for example*, *to indicate these patients for reasons of oncological tracing*, *is because we often have ages*, *protocols […]*, *I know I have to do at least one tracing ready*, *this I know”*

When the doctor says that the *ease is due to having protocols (in this case for tracing cancer)*, one has to think: can this be plausible? Having a protocol does not necessarily give meaning to the medical conduct. For a protocol to be successful, have good resolution, it is necessary to have an idea that supports it. If there are medical protocols, why aren’t there dental protocols? Perhaps this is because institutional logic follows the paradigm that is dominant there. A presupposition would be that the medical areas keep their distance from the dental areas by reason of the difference in the model determined for historical reasons.

In its turn, the head and neck surgeon or otorhinolaryngologist make this interface with oral health because they are interdependent while there are anatomopathological reasons for this.

The professional relationship of the psychiatrist with mental disease occurs through the sensorial perception that he acquired by learning about the clinical manifestations in the sphere of emotions and behavior.

**I3** *“I am a little in doubt*, *therefore*, *for example*, *this part of sialorrhea*, *for example… the tooth softening*? *if this really happens or if it’s something that happened with the two coinciding together (hypo and hypersalivation)*. *But I really feel deeply in doubt*, *and end up referring [the patient] to the dentist at the health unit*, *so that they refer the patient*.*”*

## Discussion

Medical education, the structural paradigm of which is the biomedical model, is focused on the concepts of the health-disease process in the anatomy and physiology and of the human body. This model, added to the exhausting type of/hours of work that doctor do, may lead to an organization of work in the manner of “fabrication”, which results in problems of identity for both the patient and doctor as subjects in this relationship since both are treated as parts of an organizational structure [15]. An organization that is hardly adequate for medical work will reduce the moment of clinical encounter to a mere or instrumental act [16]. So what effect will this have on the medical health-disease and health-disease relationships in Dentistry?

According to Thomas Kuhn (1970) [17] in his concept of paradigm, the model of practitioners of a science that limits their understanding of their object of study. According to this logic, in each area of knowledge there will be a concentration and union of forces around the paradigm installed in that nucleus of knowledge. The structures have a logic that limits interfaces, and non-cooperative junctions. In academic medicine, a certain existent structure inevitably becomes stronger than individual decisions. One is faced with a complex phenomenon that would demand multiple interventions, such as is the case of the psychiatric patient. The doctor is led to seek help to attend the diverse needs of patients.

The concept of paradigm used here was based on the epistemology of the sciences that Thomas Kuhn presented in his work, considered the work most cited worldwide by the human sciences. For the physicist “*a paradigm is that which the members of a community share and*, *conversely*, *a scientific community consists of men who share a paradigm*”. In this sense, when analyzing the results of the present study, we found that both subjects (doctors and dentists) were inserted into the same paradigm, the biomedical type.

However, in the continuity of Kuhnian thought, “*a scientific community is formed by the practitioners of a scientific specialty*. *They were submitted to a similar professional initiation and education*” [17] (p. 281–283). Here a paradigmatic subdivision is revealed, composed of different scientific communities, inserted into the same paradigm; in our case, there is the community formed by medical professionals, and there is another community formed by dentistry professionals, two different specialties, formed within the same paradigm.

Although the biomedical paradigm may also be dominant in dentistry, the institutional organization of medical education differs from that of the dentist. This ends up structuring the professional groups according to this organization, as though they were separate paradigms. Theoretically there are many areas of convergence, but in their respective units, the groups are distinguished, thereby reinforcing their own paradigm.

The statements that the doctor makes about *disease*; that is to say, a scientifically studied disease, are due to the medical school education he received. Whoever did not have the same education (in the same nucleus of information) would not have the same phenomenological relationship with the issue observed. The same situation occurs with dentists who have a perception of the oral health of their patients face to face with the patient, they naturally pay attention to the aspects perceived in the mouth, gums, possible abnormalities of shape, color, breath, etc. This practice arises from their education and is consolidated in the process of clinical activity. Within the same paradigm, the statement of *disease* (scientifically constituted disease) has a similar meaning. This explains why the medical residents do not understand their role when faced with the oral health of their patients, but they understand their commitment to the prevention of gynecological cancer, for example. This concerns a phenomenological perception that is expressed within the same nucleus of his education. In this case, gynecology.

It does not, therefore, concern having or not having protocols for guiding attendances. A protocol would only make sense if it had an idea that supported it; if its elaboration had previously been discussed by the nucleus of knowledge that generated it.

Another point of discomfort observed was related to the attribution of responsibility for the patient. To the extent that the doctors do not have command of the knowledge required for conducting the instructions about and/or referrals of oral problems of their patients, they could blame it on the patients themselves. Considering that their eventual instructions would not bring about the effects, supposing that the patient would not care for himself/herself. In view of the anguish of rarely dealing with the occurrence, the doctor would attribute the responsibility to the supposed culture of this population.

With regard to the second category of this study, it revealed the “nonparticipation” of the dentist in the so-called “collusion of anonymity”, which are flows that tend to occur within each area of knowledge. According to Balint, author of traditional British medical psychology, medical specialists frequently refer patients to specialists within the network of specialists, so that “all of them are doctors of the patient and none of them are the patient’s doctor [11]. There is the creation—not necessarily voluntarily—of a non-responsibility of each doctor towards a certain patient.

In practice, we know the doctors of psychiatry normally have proximity to psychologists. Other medical specialties have proximity, let us say phenomenologically, to the dentist because they a border specialty; they are those that act in close, tangential areas, such as otorhinolaryngology, oncology of the head and neck, and others, on account of the confluence of clinical diagnosis.

## Conclusions

- The training paradigm is superimposed on personal decisions, making it difficult to oppose the institutional model.
- Symptoms related to oral health did not have the relevance expected for the type of management considered adequate in a medical training environment.
- There are barriers to interdisciplinary action, which are intertwined with paradigmatic barriers.
- There was emphasis on the importance of new interdisciplinary studies between psychiatry and dentistry, considering the gaps found in the relationship between the two practices.

## Supporting information

S1 File The transcripts of the interviews were made available in full for this journal. (DOCX)
