# Supplementary material for: Perceptions of the oral health of their patients reported by Brazilian medical residents in psychiatry
Source: PLoS One. 2023 Apr 17;18(4):e0282945. doi: 10.1371/journal.pone.0282945 (PMC10109482; doi:10.1371/journal.pone.0282945)
Supplement: S1 File — (DOCX) [file pone.0282945.s001.docx]

**Áudio: LU01**

**Tempo de gravação: 00:13:17**

**Entrevistadora** – O objetivo da nossa pesquisa é entender melhor esse cenário da relação médico-profissional, para a gente compreender qual a percepção dos médicos a respeito da saúde bucal dos pacientes com transtorno mental severo.

**Sr. I1 (Entrevistado)** – Uhum.

**Entrevistadora** – Porque é uma maneira, é uma das maneiras que a gente tem de poder interferir um pouco nesse cenário, futuramente, com algum projeto de intervenção, porque a gente sabe que a condição bucal desses pacientes é muito pior do que da população em geral. Então a ideia é essa. E aí eu queria saber de você como é que você acha que costuma ser a percepção dos médicos em geral em relação à saúde bucal dos pacientes com transtorno mental? Vocês têm essa atenção, é impossível isso, dada as enormes outras demandas do paciente? Como é que isso se dá na sua opinião, I1?

**Sr. I1 (Entrevistado)** – Eu acho que depende muito do quadro que a gente estiver lidando. Tem uns quadros que chamam muito a atenção para essa dimensão de higiene, fico pensando aqui no paciente com transtorno obsessivo compulsivo, muitas vezes o elemento da higiene, do tempo, é uma coisa que a gente ativamente pergunta. E tem alguns pacientes que eu pergunto: tem escovado os dentes, alguns ou porque escovam demais ou escovam de menos, eu tenho problemas em cada caso, é claro. Então surge isso aí, alguns outros pacientes, por exemplo, transtorno alimentar, eu já não tenho tanta prática, mas sei que é uma coisa que você tem que ficar atento, porque tem deterioração, tem outras coisas. Já outros pacientes com transtorno mental grave, tipo esquizofrenia, transtorno afetivo bipolar, olha, muitas vezes são coisas que ou passam desapercebidas.

**Entrevistadora** – É, imagino que sim, diante do quadro todo.

**Sr. I1 (Entrevistado)** – Ou até mesmo quando é uma coisa muito, muito até pertinente, são quadros que a gente já está conseguindo fazer malabarismo, já fica tão contente com o malabarismo que faz, por exemplo, tolerante até com o paciente que está fumando, o paciente que está tendo uma série de outras questões, mas está conseguindo manter algum grau de estabilidade, principalmente da esquizofrenia, que tem muito paciente com a condição imagino que odontológica ruim, mas que haja vista toda a gravidade, a gente é tolerante por assim dizer.

**Entrevistadora** – Sim, sim. Até por que eu imagino que eles tenham alguma... Que eu imagino que eles tenham disfunções. Desculpa, deixa eu só te explicar, são minhas alunas, elas estavam me procurando. Dr. I1, Bruna, Júlia, você se importa que elas estejam?

**Sr. I1 (Entrevistado)** – Não, só não vou ter cadeira para vocês, se quiserem sentar ali.

**Entrevistadora** – Eu não achei que elas chegariam, que elas estavam tentando vaga e não conseguiam, por aí. Então, I1, eu imagino até que esses pacientes tenham dificuldades alimentares outras, não é? Os esquizofrênicos, por exemplo, transtorno bipolar, tem que lidar com outras questões interligadas à boca, não? Como dificuldades alimentares, tabagismo.

**Sr. I1 (Entrevistado)** – É, são pacientes que às vezes têm dificuldade para tomar o remédio, quanto mais negociar a questão alimentar. Os quadros que estão mais estáveis a gente tenta, pensando no ponto de vista metabólico, as medicações que a gente dá mexem muito com o colesterol. Mas acaba que é mais essa dimensão, admito que não vem tanto, acho que essa preocupação, essa proatividade talvez na questão da higiene bucal para esses especificamente.

**Entrevistadora** – Compreensível.

**Sr. I1 (Entrevistado)** – Mas uma coisa que às vezes coloca a gente até para pensar um pouco, porque são pacientes que ficam muito desassistidos e sobra para o psiquiatra às vezes fazer alguns encaminhamentos. Então é muito comum, por exemplo, eu encaminhar paciente com esquizofrenia pela primeira vez para o ginecologista.

**Entrevistadora** – Nossa.

**Sr. I1 (Entrevistado)** – Que ele tem esquizofrenia, nunca foi para o ginecologista, porque o problema sempre foi esquizofrenia, mas é toda uma outra dimensão que precisa ser vista e tudo mais. Já para dentista admito que estava até a se pensar, não é tão frequente.

**Entrevistadora** – Na verdade, não é um negócio que nós já tínhamos, mesmo, isso é compreensível de todas as outras demandas, não é?

**Sr. I1 (Entrevistado)** – Sim.

**Entrevistadora** – Agora a nossa ideia é criar algum caminho para facilitar isso para vocês, inclusive, não é, talvez facilitar o acesso deles, enfim, e uma série de ideias aí, não é.

**Sr. I1 (Entrevistado)** – Eu fiquei pensando agora, não sei, porque você fala transtorno mental grave, eu penso esquizofrenia e transtorno efetivo bipolar. Mas já tive até algumas experiências no autismo.

**Entrevistadora** – Ah, perfeito, perfeito.

**Sr. I1 (Entrevistado)** – Com criança e adolescente, mais comumente, aqui pelo menos é uma prática da Unicamp, porque é toda uma questão, não é, para a criança com autismo tolerar, aceitar passar pelo dentista.

**Entrevistadora** – Não tenha dúvida, para nós é um desafio gigante.

**Sr. I1 (Entrevistado)** – Então, e aí é uma coisa que às vezes eu já até fiquei com dúvida de encaminhamento, mães dizendo que o dentista que elas vão, não consegue, queria saber se tem um lugar específico. Acaba encaminhando para cá, mais por uma ideia acho que meio fantasiosa de que aqui na Unicamp o pessoal, odonto, vai ter alguém que vai saber mexer com isso. Mas assim, nem sei se é o fluxo mais adequado, sendo bem sincero.

**Entrevistadora** – É, na verdade, você está fazendo o certo, você está encaminhando corretamente. É desafiador para nós, mas a gente tem que, tem algumas estratégias para a gente poder lançar mão, e na verdade, é um grande problema, até por que muitas crianças estão aí na troca da dentição, a hora h da intervenção, hora boa de intervir, a hora necessária, na verdade, de intervir. E é um grande problema, porque de fato a mãe acaba deixando, porque a mesma coisa, tantas outras demandas.

**Sr. I1 (Entrevistado)** – Escolher as demandas.

**Entrevistadora** – Isso, escolher as batalhas, interessante. E assim, em relação a eles, você percebe eles reclamando da situação bucal como uma demanda deles ou não necessariamente?

**Sr. I1 (Entrevistado)** – Não. Não, recordando aqui, eu não lembro de nenhum paciente trazendo essa queixa.

**Entrevistadora** – Acaba sendo menos importante, não é, eu imagino.

**Sr. I1 (Entrevistado)** – Acho que já tive um paciente que se queixou de uma halitose, mas uma coisa que foi relativo de manejo. Mas não, eles trazendo, não. Já tive um paciente também aqui tentando puxar de memória bem, que teve uma questão, por exemplo, paciente de quadro depressivo, ocioso, quadro de dor dentária, também já estava com sem maiores problemas, assim. O encaminhamento foi simples. Mas agora pacientes com esquizofrenia e transtorno bipolar, não, realmente, não.

**Entrevistadora** – E quando você fala de outras prioridades, conta um pouco para a gente algumas delas, assim, eu entendo que haja, mas queria só entender um pouco melhor de outras prioridades antes da...

**Sr. I1 (Entrevistado)** – Sim, sim. Que eu acho que tem dois atendimentos mais frequentes aqui, pelo menos no ambulatório, que são os pacientes que estão em surto, em crise, e às vezes vem a coisa de garantir a segurança deles, garantir a segurança dos outros, garantir a ingesta da medicação correta. Enfim, essas situações que às vezes são mais urgentes, até mais do que qualquer outra questão de saúde clínica em alguns momentos. E o outro paciente que está estável e a gente tem que lidar com sintomas especiais e sintoma negativo, não é?

**Entrevistadora** – Aham.

**Sr. I1 (Entrevistado)** – São pacientes mais apáticos, menos interessados, menos proativos. E esses, digamos, tem que ter toda uma energia, uma proatividade para estimular ele e a família, colocar especialmente em alguma atividade integradora, seja atividade física, seja ocupacional.

**Entrevistadora** – Que já deve ser um baita desafio.

**Sr. I1 (Entrevistado)** – Um desafio.

**Entrevistadora** – Imagino.

**Sr. I1 (Entrevistado)** – Especialmente a parte de atividade física, porque a gente sente que as nossas medicações estão por trás de aumentar a sonolência, aumenta colesterol, diminui a expectativa de vida. Então estou com aquela consulta que eu tenho um tempo, vou tentar fazer uma conscientização com o paciente, muitas vezes o que eu vou focar é a questão da atividade física, atividades socializantes. Acaba que é o mais frequente. E quando surgem demandas clínicas no paciente, aí a gente tenta ir pontuando e tenta fazer os encaminhamentos, tenta fazer os arranjos que der para fazer. É só pensando assim, admitindo bem, eu acho que foi só esses casos de ida para ginecologista que nunca teve, que eu fui fazendo os encaminhamentos.

**Entrevistadora** – Entendi.

**Sr. I1 (Entrevistado)** – E assim eu fui fazendo. E um outro caso de paciente com mais de 50 anos e algumas queixas para rastreio de câncer.

**Entrevistadora** – Ah sim, perfeito.

**Sr. I1 (Entrevistado)** – Isso aí eu já fiz também alguns encaminhamentos nesse sentido. Em busca de sintomas, essas coisas, sabendo que era um paciente menos atuante na rede, menos demandante dos direitos e eu acabei fazendo isso algumas vezes.

**Entrevistadora** – Sim, perfeito. Muito bom, foi bem rico. Eu acho que tem mais alguma coisa que você queira acrescentar a respeito desse assunto que você acha...

**Sr. I1 (Entrevistado)** – Com certeza me faz pensar, de pensar como é que poderia abordar melhor essas questões, e que tipo talvez, não sei, de sinais de alarme, alguma coisa assim pontual que eu pudesse dizer: opa, isso aqui é uma indicação.

**Entrevistadora** – Ótimo, é isso que a gente quer.

**Sr. I1 (Entrevistado)** – Porque eu fico sem saber, será que é só, porque na intervenção tem pouquíssimo, quase nada de saúde bucal. Acho que eu tive uma ou duas aulas, talvez.

**Entrevistadora** – Uhum.

**Sr. I1 (Entrevistado)** – E aí o que é que eu faço, o paciente está com pouca escovação, o paciente está com dor, o paciente que está com aspecto dentário ruim, paciente que tem halitose ou não, enfim, não sei.

**Entrevistadora** – Num bate-papo assim rápido, impossível te passar alguma coisa, mas acho bem oportuno pelo que eu estou entendendo que você fala e que eu também percebo, é oportuno que houvesse um treinamento ainda que rápido para vocês poderem ter essa noção inicial e poder encaminhar. O encaminhamento é, mas depende o local onde você está, no caso aqui o paciente para cá, mesmo para uma unidade de saúde da família, onde ele reside, se não tiver unidade de saúde da família, enfim, unidade básica de saúde do território onde ele reside, esse é o encaminhamento. E os sinais são os visíveis para vocês.

**Sr. I1 (Entrevistado)** – Estado de deterioração.

**Entrevistadora** – Deterioração, cárie e a gengiva, quanto mais vermelha e brilhante, mais ela precisa de ajuda. A gengiva ao contrário da gente, a gengiva quanto mais pálida, menor é a possibilidade dela ter uma doença gengival. Brilhante, sangrante, brilhante precisa de ajuda.

**Sr. I1 (Entrevistado)** – Eu fico pensando que a facilidade que eu tenho, por exemplo, para indicar esses pacientes por motivos de rastreio oncológico, é porque a gente muitas vezes tem idades, protocolos muito, eu sei que é (ininteligível), tem que fazer pelo menos um rastreio pronto, isso eu sei. Porque às vezes até um questionário de duas perguntas, eu sei que na unidade básica quase nunca fazem o de duas perguntas para depressão, uma coisa que passa pouco. Mas o pessoal sabe, rastreio de câncer uma coisa que já está padronizada.

**Entrevistadora** – Isso, perfeito.

**Sr. I1 (Entrevistado)** – Só duas perguntas, não é, o pessoal acaba deixando passar. Mas câncer não, todo mundo sabe, aos 50 anos tem que fazer tal, mulher a partir de tal idade faz tal. Talvez tenha um proxy desses, facilitasse um pouco.

**Entrevistadora** – Ótimo, ótima ideia.

**Sr. I1 (Entrevistado)** – Se eu disser que todo paciente precisa a cada sei lá, x anos fazer y avaliações dentárias.

**Entrevistadora** – Perfeito.

**Sr. I1 (Entrevistado)** – Aí fica talvez uma coisa mais na cabeça.

**Entrevistadora** – Ótimo, bem objetivo isso, já é uma ótima ideia. Muito bom.

**Sr. I1 (Entrevistado)** – Que eu fico pensando, para mim ajudaria isso.

**Entrevistadora** – Sim, ótimo. Muito bom. I1, muito obrigada, desculpa aí a nossa correria, porque hoje acabou sendo um dia muito corrido. Eu não sei como é que finaliza.

**Áudio: LU02**

**Tempo de gravação: 00:12:17**

**Entrevistadora** – Eu estava te perguntando como é que você vê, onde você acha que os colegas psiquiatras, os psiquiatras em geral veem essa questão da saúde bucal dos pacientes com transtorno psiquiátrico.

**Sr. I2 (Entrevistado)** – Eu acho que não só o especialista, mas a medicina de forma geral, não tem esse cuidado com a saúde bucal. Desde a formação assim, a gente não tem essa, uma matéria que vá contemplar isso. Assim, a gente acredita assim: ah, tem alguma alteração, o dentista tem uma facilidade de acesso, eu costumo encaminhar para o dentista quando vê alguma coisa desse tipo. Em se tratando de pacientes na psiquiatria, o que é que eu vejo, existe o perfil de paciente de uma população mais pobre que usa o SUS, e pacientes particulares e tudo. A gente nota não só na psiquiatria, mas em todas as áreas desses pacientes que tem uma precariedade maior, não é?

**Entrevistadora** – Uhum.

**Sr. I2 (Entrevistado)** – Eu acredito assim, falta de informação deles, eles ainda têm aquela questão de antigamente que o dente estragado é para extrair, não é?

**Entrevistadora** – Sim.

**Sr. I2 (Entrevistado)** – Não fazem canal, não fazem intervenção nenhuma para tentar salvar aquele dente, é mais extração, não tem essa preocupação tão grande. A maioria deles preferem extrair os dentes e colocar prótese depois. O que é que a gente percebe assim, então, dentro da psiquiatria, é que eu acho que de uma maneira geral, é uma coisa assim que por mais que a gente encaminhe, não tem um acompanhamento adequado, entendeu, quando você nota.

**Entrevistadora** – Nos encaminhamentos que vocês fazem de outras questões?

**Sr. I2 (Entrevistado)** – Sim, um paciente do SUS que tem uma precariedade da higiene bucal, e a gente encaminha, ele muitas vezes não adere, não vai, porque eles não preocupam mesmo com isso, sabe, para eles, eles não entendem que o dente é um, para eles parece assim que eles acham que o dente é uma coisa já morta assim, não osso dentro da boca.

**Entrevistadora** – Entendi.

**Sr. I2 (Entrevistado)** – Que vai perdendo, depois perde e coloca uma prótese. Eles não pensam de saúde bucal, os pacientes.

**Entrevistadora** – E você acha que por que, falta de conhecimento ou é cultural?

**Sr. I2 (Entrevistado)** – Eu acho que é cultural, eu acho que assim, muitos desses pacientes viveram uma época de dentista prático. Então eu falo que minha esposa é dentista.

**Entrevistadora** – Ah é?

**Sr. I2 (Entrevistado)** – É. E ela trabalhou muito tempo na saúde básica.

**Entrevistadora** – Sim, sim.

**Sr. I2 (Entrevistado)** – E ela fala essa questão, desde a escovação, de não saber escovação, passar fio dental. Tudo isso é uma coisa que deveria ser muito melhor na saúde bucal. Eu acredito que assim, futuramente essas crianças que hoje tem esse contato, pode ser que tenham essa orientação no futuro, de se preocupar. Mas essa população que não teve isso, essa educação da saúde bucal, eu acredito que essas pessoas que tem mais dificuldade, sabe. Eu falo até mesmo assim, minha esposa me ensinou muito, eu médico e tudo mais, eu brinco com ela que até pouco tempo atrás eu achava que assim, o ciclo da vida era você perder os dentes e usar a prótese no final da vida. Até por que meus avós eram assim, então eu via aquilo, falava ah.

**Entrevistadora** – Como natural.

**Sr. I2 (Entrevistado)** – Perder os dentes.

**Entrevistadora** – Sim, sim.

**Sr. I2 (Entrevistado)** – E assim, a minha sogra é dentista e a família toda assim, aí eles falam muito, eles ficam muito indignados com isso. Tem outros países também que a gente vê, a gente teve na Argentina e vê que a população lá também possui tratamento caro, não tem igual tem aqui. Mas aí tem essa questão, a pessoa tem mais prioridades do que cuidar dos dentes.

**Entrevistadora** – Sim, eu fiquei um mês, dois meses aqui só observando e pude perceber também que há uma gama gigante de prioridades dos pacientes psiquiátricos também, eu também como dentista também não tinha ideia do tanto de outras questões graves que eles têm, não é, então para mim também foi um ensinamento. E aí entrar a saúde bucal nesse quadro de prioridades, como é que você vê isso? Você acha que é uma necessidade ou de fato realisticamente falando, isso de fato não cabe aí?

**Sr. I2 (Entrevistado)** – É uma necessidade, com certeza, porque assim, a gente sabe o prejuízo que uma higiene precária pode causar, não é?

**Entrevistadora** – Uhum.

**Sr. I2 (Entrevistado)** – A gente sabe, assim, a gente vê casos extremos, que o paciente pode ter infecção.

**Entrevistadora** – Pois é, essa é a questão.

**Sr. I2 (Entrevistado)** – Não vejo a proatividade do paciente para isso, entendeu?

**Entrevistadora** – Entendi.

**Sr. I2 (Entrevistado)** – Não sei se é por que a gente não faz uma promoção adequada, a gente costuma falar muito de psicoeducação, que a gente tenta convencer o paciente de algumas coisas, através da habilidade da psicoeducação. Poderia de alguma maneira ter alguma psicoeducação nesse sentido, através de seja de palestra, alguma coisa para mostrar para o paciente a importância disso. Para esse paciente, eu acho que a maioria são muito sem instrução.

**Entrevistadora** – Uhum.

**Sr. I2 (Entrevistado)** – Mas mesmo assim eu acho que assim, nós mesmos, eu, marido de dentista, não rotineiramente como deveria, a gente acaba de modo geral indo ao dentista quando tem uma dor de dente.

**Entrevistadora** – Dor, uma urgência, quebrou um dente.

**Sr. I2 (Entrevistado)** – Só para urgência. Então essa parte profilática é muito (ininteligível), ainda mais para esse perfil de paciente.

**Entrevistadora** – Você tem visto assim casos que te chamam a atenção, ou que eles tenham relatado o sofrimento com saúde bucal? Dor, tudo muito estragado, enfim, que chega até ser aparente?

**Sr. I2 (Entrevistado)** – Olha, nesse tempo que eu estou na psiquiatria, nunca tive uma queixa.

**Entrevistadora** – Não? Ah tá.

**Sr. I2 (Entrevistado)** – Deles reclamarem, que você vê que está ruim, mas para eles.

**Entrevistadora** – E quando você vê que está ruim, o que é que te causa isso, a você, médico?

**Sr. I2 (Entrevistado)** – Então, como eu não, assim, eu tenho dom para odonto, acaba que a gente fica assim, a gente meio que parece que se torna normal, entendeu?

**Entrevistadora** – Entendi.

**Sr. I2 (Entrevistado)** – Que a maioria tem a precariedade dos dentes, acaba que vira um cotidiano mesmo. Fica comum, você para de notar tanto.

**Entrevistadora** – Entendi.

**Sr. I2 (Entrevistado)** – Porque não é tanto o seu foco de atenção também. Enxugar gelo também, não é, dessa questão de tentar uma coisa, e saber assim que o máximo que vai acontecer é extrair os dentes todos e colocar prótese. Não tem nada que eu veja assim tão promissor, sabe, para isso.

**Entrevistadora** – Sei.

**Sr. I2 (Entrevistado)** – Então fico triste, porque é ruim.

**Entrevistadora** – Claro.

**Sr. I2 (Entrevistado)** – Mas não vejo nada assim resolutivo. Não tenho nenhuma visão sobre o que pode, poderia resolver. Eu acho que vai ser a longo prazo que os dentistas estão fazendo plano de governo hoje em dia, eles têm essas crianças aí para ver.

**Entrevistadora** – Que tem outras rotinas que acho que talvez por estarem mais estabelecidas, como, por exemplo, o Papa Nicolau e outras questões, que acho que vocês acabam perguntando e encaminhando, porque existe um protocolo para isso, então da odontologia não tem, pode ser isso também, você acha uma questão? A gente não tem um protocolo que diga: aos tantos anos tem que fazer, a gente até tem, mas acho que isso não é tão divulgado para vocês médicos como você mesmo disse, passou dos 60, por exemplo, tem que fazer exame bucal contra o câncer. E coisa que o Papa Nicolau, por exemplo, mamografia aos 50 anos vocês têm um protocolo aí que diz que tem que fazer, enfim, pode ser isso, você acha que também tem?

**Sr. I2 (Entrevistado)** – Com certeza.

**Entrevistadora** – Talvez, é uma hipótese, também estou lançando essa hipótese agora.

**Sr. I2 (Entrevistado)** – Eu acredito que sim, falta isso.

**Entrevistadora** – Uma coisa estabelecida, não é?

**Sr. I2 (Entrevistado)** – A gente só vai preocupar quando é alguma coisa assim cólica, alguma coisa assim que salta aos olhos.

**Entrevistadora** – Que salta aos olhos, pode ser isso.

**Sr. I2 (Entrevistado)** – Não é uma coisa de investigar, igual o Papa Nicolau é bem esclarecido, a gente vai lá, antes de a lesão aparecer, você já tem medidas para fazer, para ver aquilo ali.

**Entrevistadora** – Sim.

**Sr. I2 (Entrevistado)** – Na parte da odonto eu não sei como é que funciona, nunca tive essa experiência.

**Entrevistadora** – Está certo. Eu acho que você me traz algumas, uns subsídios bem importantes até para a gente fazer propostas. É interessante, mesmo. Eu acho que é isso. Você tem mais alguma coisa que você queira acrescentar sobre isso?

**Sr. I2 (Entrevistado)** – Não, acredito que infelizmente está um pouco distante da nossa realidade.

**Entrevistadora** – Da realidade.

**Sr. I2 (Entrevistado)** – Eu acredito, não sei, nunca trabalhei em CAPS, não sei se você já foi no CAPS.

**Entrevistadora** – Sim, conheço, não trabalhei também, mas conheço. Depende, em geral não, em geral eles encaminham para o dentista da atenção básica. Mas até pode ter, nada impede que haja um dentista que vá lá no CAPS ou que se estabeleça lá também. Eu não conheço CAPS que tenha dentista dentro dele, não é, mas não significa que não tenha, eu que não conheço. Mas em geral, os CAPS eles encaminham o paciente para fazer o tratamento odontológico na atenção básica. Mas é isso.

**Sr. I2 (Entrevistado)** – Mas por que o que eu acho que vai acontecer mais, seria de na especialidade mesmo, da odontologia tentar ser inserida. Mostrar que tem coisas que são muito importantes, que é uma oportunidade de inserir de fato coisas que às vezes seja uma oportunidade única, o paciente não vai em outro lugar para conseguir.

**Entrevistadora** – Sim. Por isso que eu comecei por aqui, porque o maior contato deles é com vocês, vocês psiquiatras, eu acredito que o contato mais constante deles, não é.

**Sr. I2 (Entrevistado)** – Aham.

**Entrevistadora** – Ótimo. Muito obrigada, I2, I2, não Rodrigo, desculpa.

**Sr. I2 (Entrevistado)** – Aí depois você traz o termo para eu assinar.

**Entrevistadora** – Eu trago sim, I2, foi uma falha, eu saí correndo, estava com medo de te atrasar. Vou pegar minha carteira aqui. Eu trago e deixo aí com você então. Obrigada, viu.

**Áudio: LU03**

**Tempo de gravação: 00:06:36**

**Entrevistadora** – Então assim, a gente queria, a nossa pesquisa ela busca entender qual a percepção, o significado que os médicos têm a respeito de saúde bucal dos pacientes com transtorno mental. E nesse sentido eu queria saber, vocês têm uma prática que eu já fiquei aqui num semestre me aculturando, uma prática de atendimento intenso, não é, atende bastante. E como é que você acha que é assim na percepção dos médicos em geral, como é que eles veem a questão da boca do paciente com transtorno mental?

**Sr. I3 (Entrevistado) –** Vou ser bem honesto, é uma coisa que eu acho que é um pouco negligenciada, a parte da saúde bucal. Porque muitos pacientes são muito debilitados e acabam os cuidadores dando conta disso, o cuidador da higiene bucal e toda essa parte. Em outros pacientes apresentam problemas de saúde bucal que a gente acaba não conseguindo dar muita atenção.

**Entrevistadora** – Uhum.

**Sr. I3 (Entrevistado) –** Tem paciente que por causa de muitos remédios, e isso acaba afetando muito a saúde bucal.

**Entrevistadora** – Sim.

**Sr. I3 (Entrevistado) –** E a gente acaba não sabendo muito como indicar ou o que fazer para falar sobre isso. E além do mais, também tem outros pacientes que ficam com a boca seca, então acabam gerando muita lesão na boca.

**Entrevistadora** – Ainda tem isso, não é, da boca seca, medicações que causam xerostomia?

**Sr. I3 (Entrevistado) –** Tem. Tem muitos remédios, principalmente os antidepressivos tricíclicos, xerostomia. Aí acaba dando úlcera bucal.

**Entrevistadora** – Não, é terrível, xerostomia para nós é um mal terrível, que abre portas para uma série de coisas, para cárie, para doença periodontal, é um problema, mesmo. E assim, deles, vocês notam queixas deles, dos pacientes a respeito ou não, são tantas queixas, são tantos problemas, não é?

**Sr. I3 (Entrevistado) –** Eu noto algumas queixas assim, então principalmente eu tenho uma paciente que reclama muito da hipersalivação e sente que os dentes dela estão amolecendo por causa da (ininteligível). E também tem o procedimento DCP, que a gente faz na enfermaria, teve já uma paciente esse ano que quebrou o dente.

**Entrevistadora** – Não existe uma proteção, não é? Tem, mas mesmo assim pode acontecer?

**Sr. I3 (Entrevistado) –** Pode acontecer. E aí ela acabou quebrando o dente frontal. Mas ela, mas assim, que existem queixas que o paciente nunca se queixa, tem muita dor no dente também, isso mais na enfermaria, aparece muita dor de dente. Mas a gente acaba o chamamento da consulta da odontologia.

**Entrevistadora** – Ah, vocês têm agora aqui, não é?

**Sr. I3 (Entrevistado) –** Sim. Tem consulta, mas eles não conseguem atuar tanto lá em cima. É muito limitado os procedimentos que eles conseguem fazer.

**Entrevistadora** – Mas vocês estão fazendo a parte de vocês, pelo menos chamar, não é?

**Sr. I3 (Entrevistado) –** Sim.

**Entrevistadora** – Eu imagino que sejam muitas prioridades desses pacientes, muitas demandas que eles devem trazer para além do problema em si, tudo que o problema causa, não é?

**Sr. I3 (Entrevistado) –** Sim.

**Entrevistadora** – Eu imagino.

**Sr. I3 (Entrevistado) –** Muita demanda assim, a maior dificuldade mesmo é como falei, são os pacientes mais graves. Que eles não cuidam da própria higiene bucal, e o cuidador tem que forçar às vezes quando eles não entendem por que é que tem que cuidar, e aí acaba apresentando muitas lesões na boca, o dente acaba ficando bem, aparece cárie.

**Entrevistadora** – Entendi. E assim, quando eles trazem demandas, como é que você se sente, como é que é para você?

**Sr. I3 (Entrevistado) –** Eu fico um pouco em dúvida, então, por exemplo, essa parte da ação é evidente, isso acontece mesmo, isso é uma coisa que aconteceu de coincidir os dois juntos. Mas eu fico muito em dúvida, eu acabo encaminhando para a odontologia do posto, para eles encaminharem.

**Entrevistadora** – Isso, está correto, é isso mesmo. Na verdade, assim, o excesso da saliva via de regra não era para ter problemas, não é, quanto mais irrigado ali, menor a chance de, só que o problema é o seguinte, conforme tem essa sensação, esse incômodo para eles, menos eles vão cuidar, também tem isso. Então uma coisa leva a outra, sem contar que a sialorreia geralmente causa queilite também, não é?

**Sr. I3 (Entrevistado) –** Isso.

**Entrevistadora** – Muita queilite angular, e aí fica mais difícil escovar porque dói. Então como tudo para eles, acho que vira uma cadeia de problemas, não é, um problema leva ao outro. Basicamente isso. Ah, a ideia da gente é tentar entender melhor essa relação para daí provavelmente depois da pesquisa, fazer alguma proposta de intervenção que seja protocolar ou pontual, que seja, para vocês poderem, para facilitar a vida de vocês em relação a isso também.

**Sr. I3 (Entrevistado) –** Acho que na verdade, há um pouco da minha parte, não sei dizer dos residentes, um pouco de educação na área assim. Eu aprender um pouco mais sobre isso.

**Entrevistadora** – Sim, seria interessante.

**Sr. I3 (Entrevistado) –** Para eu saber como lidar, como falar com os pacientes, porque assim, para sialorreia a gente usa atropina, tudo bem. Mas para xerostomia...

**Entrevistadora** – Saliva artificial é difícil para eles.

**Sr. I3 (Entrevistado) –** Exatamente. Então o que eu tenho indicado para os pacientes normalmente é ficar com uma garrafinha d'água, ficar molhando de 15 em 15 minutos, mas eu não sei se é o procedimento correto, não é.

**Entrevistadora** – Até é, está hidratando, mas o problema é eles fazerem também.

**Sr. I3 (Entrevistado) –** É, exatamente. Então assim, seria mais para eu saber como lidar com essas situações que vão aparecendo para a gente, e quando indicar direto para o cirurgião odontológico, como indicar para o posto de saúde.

**Entrevistadora** – Ah, perfeito. Isso já é uma dúvida importante.

**Sr. I3 (Entrevistado) –** Para onde indicar.

**Entrevistadora** – Vai para o especialista ou vai para o clínico geral.

**Sr. I3 (Entrevistado) –** Por exemplo, de acordo que eu mando aqui para cima que é mais especializado, que a gente tem a odontologia aqui em cima, para quando manda para o posto.

**Entrevistadora** – Perfeito. Já é uma dúvida importante para a gente pensar e traçar um caminho, não é. Muito bom. Tem mais alguma coisa que você queira acrescentar em relação ao assunto?

**Sr. I3 (Entrevistado) –** Eu acho que é só isso mesmo.

**Entrevistadora** – Está ótimo. I3, muito obrigada pela sua contribuição.

**Sr. I3 (Entrevistado) –** De nada.

**Entrevistadora** – A gente veio, eu cometi um erro hoje, deixei meu carro estão longe...

**Áudio: LU04**

**Tempo de gravação: 00:08:26**

**Entrevistadora** – Então I4, a gente gostaria de saber como na sua opinião é a percepção de saúde bucal dos médicos em geral que atendem pacientes com transtorno mental? Do psiquiatra que atende o paciente com transtorno mental?

**Sr. I4 (Entrevistado)** – Você diz de uma maneira geral?

**Entrevistadora** – É, de uma maneira geral?

**Sr. I4 (Entrevistado)** – Acredito que seja pobre (ininteligível), na verdade. A gente aqui internamente aborda na verdade, nas consultas. E mesmo vendo a própria dentição dos nossos pacientes, seja no pronto-socorro, na enfermaria ou aqui, a gente vê que é marcadamente assim bem malcuidado. E assim, não conheço ninguém na verdade, por exemplo, algum caso que a gente tenha encaminhado para avaliação odontológica, acho que nesse sentido é bem ruim na verdade.

**Entrevistadora** – E assim, na sua opinião, por que é que você acha que isso acontece?

**Sr. I4 (Entrevistado)** – Digamos assim, na população, vamos dizer assim, mais mentalmente grave, acho que são várias questões. Uma: por jeito de atender ambulatorialmente, a gente é muito focado na patologia, na questão clínica, que já por si já demanda muito. Segunda coisa, é uma coisa, eu acho que isso é uma característica dos pacientes graves mentalmente, de transtornos mentais graves, é que a gente já tem que assumir também outros papéis além do nosso. Como clínico geral, entre outras coisas relacionadas ao estigma da patologia. Então muitas vezes há uma diminuição dessas queixas que são muitas vezes comuns na população, mas do doente mental elas são bem minimizadas, acaba ficando para a gente, nós que somos psiquiatras, até fazer o manejo clínico de muitos em condições básicas. Isso faz com que a gente fique bastante sobrecarregado nesse sentido, a gente não consiga dar atenção devida a todas essas questões que vêm. A gente acaba sendo só um ponto de referência. Mas acho que é muito isso, parte de uma maneira geral de uma negligência do sistema, de como a gente vê e cuida de um paciente de saúde mental grave, transtorno bipolar, esquizofrenia. Enquanto que a gente está sobrecarregado com várias funções além de psiquiatra, por exemplo. Acho que isso é bem refletido na redução da mortalidade, a redução da expectativa de vida nesse grupo, no mínimo 10 anos. E a maior parte das causas são cardiovasculares. São doenças tratadas, claro que tem a questão dos antipsicóticos, mas de uma maneira geral ela não olhar, ela não cuidar. E muitas vezes esses pacientes em si, eles perdem a autopercepção do próprio cuidado do corpo. Então eles exigem que uma equipe muitas vezes esteja indicando para eles, percebendo eles, dando essa devolutiva. O que é bastante trabalhoso também, que exige muita organização de equipe.

**Entrevistadora** – E você nota, citou no início uma condição bucal, desfavorável, isso é notável para vocês?

**Sr. I4 (Entrevistado)** – Bastante, bastante, eu tenho pacientes só de ao abrir a boca assim, você vê que a dentição está muito prejudicada. Eles têm hábitos até eu acho que talvez pensando principalmente no esquizofrênico de perda de autocuidado, tabagismo muito intenso e hábitos alimentares também mais precários.

**Entrevistadora** – E como é que é para você se deparar com esse quadro? O que é que causa em você?

**Sr. I4 (Entrevistado)** – Ah, me causa uma sensação ruim, de algo de um pouco de fracasso, eu tenho esse tipo de sentimento quando eu vejo no sentido de ver quanto esse paciente passou por várias instâncias de cuidado e talvez ninguém tenha pensado ou encaminhado. Às vezes até um ou outro eu cheguei a comentar, conversar, na verdade, mas aí a familiar falou: não, ela é muito resistente, não quer ir. Está bom, também não tenho o que assim, como ir muito além disso.

**Entrevistadora** – A presença de dentista aqui, existe alguma, quando vocês querem encaminhar, pretendem encaminhar, tem alguma, algum contato, alguma referência aqui?

**Sr. I4 (Entrevistado)** – Não, geralmente a gente faz um encaminhamento para a unidade básica de saúde. Mas lá na enfermaria esse ano que tem o pessoal da odonto que acaba vendo o paciente. Mas só a partir desse ano, ano passado quando eu passei lá, não tinha, daí eu tinha que chamar, fazer uma interconsulta que não foi uma ou duas vezes, a gente pede com uma certa frequência. E eles vêm.

**Entrevistadora** – Agora tem também?

**Sr. I4 (Entrevistado)** – Agora tem.

**Entrevistadora** – E eles não chegam com a queixa, normalmente? O paciente?

**Sr. I4 (Entrevistado)** – Raramente, nunca escutei na verdade. Aqui no hospital, não, nunca vi. Lá em cima mais intercorrência ou coisas que a gente nota. Mas a queixa ativa, não.

**Entrevistadora** – E em termos de intercorrência, você lembra de alguma?

**Sr. I4 (Entrevistado)** – Teve duas, eu acho que ano passado, de quebrar o dente, mesmo, pela realização da eletroconvulsiva, ia fazer a restauração. Acho que essas foram as duas, e tinha alguma outra que tinha alguma realização álgica, mesmo, que a gente pensou uma avaliação.

**Entrevistadora** – Que a eletroconvulsiva fica no laboratório?

**Sr. I4 (Entrevistado)** – Isso, fica no centro cirúrgico, mesmo usando a proteção, muitas vezes acontece. Justamente até esses dois pacientes, a gente já tinha notado o quão ruim era a condição prévia da arcada, prévia ao procedimento, e que mesmo com o protetor de borracha. Ainda mais pacientes que geralmente são super (ininteligível), os dois eram depletados nutricionalmente assim, bem consumidos.

**Entrevistadora** – E tem alguma perspectiva, você imagina algum cenário que pudesse melhorar, pudesse enfim, modificar? Você relatou que é uma situação que você sente tristeza em ver, não é?

**Sr. I4 (Entrevistado)** – Aham.

**Entrevistadora** – Algo que você sugerisse ou que enfim?

**Sr. I4 (Entrevistado)** – Eu acho que esse tipo de cuidado passa muito por uma mudança estrutural, não só individual de educação individual, sabe. Das equipes de uma maneira geral, porque cuidado básico de dentição e tudo, passa muito a atenção primária em si. Claro, a gente acaba pegando aqui as consequências de algo muito avançado. Então acho que é uma questão estrutural da maneira do cuidado, e também as equipes saberem o que é pacientes esquizofrênicos, eles são, eles têm peculiaridades, eles provavelmente não vão fazer procura ativa, são coisas que podem ser abordadas de uma maneira educacional, educação da família, entre outras coisas. Mas acho que muda um pouco, tem que mudar na questão da mentalidade dos profissionais de saúde. Individualmente, claro, a gente sabe, porque aqui pelo menos é evidente, a gente bate o olho e vê. Então não é uma questão de falta de notar, talvez seja uma questão de ou quando chega já muito grave ou essas intervenções que partem de profissionais não médicos. Eu acho que os médicos também da atenção primária, eu acho que de uma maneira global.

**Entrevistadora** – Ótimo. Muito bom, acho que isso representa bastante para a gente, tem possibilidades de extrair coisas importantes. E te agradeço, queria saber se você tem alguma coisa que você queira acrescentar?

**Sr. I4 (Entrevistado)** – Não, não tenho, não. Eu que agradeço.

**Entrevistadora** – Basicamente é isso. Obrigada.

**Sr. I4 (Entrevistado)** – Eu que agradeço.

**Entrevistadora** – E, sobretudo, obrigada aí com a ajuda.

**Áudio: LU05**

**Sr.I5 (Entrevistado):** Tá...tá tudo bem.

**Entrevistadora** –Eu... preciso gravar nossa conversa para eu transcrever depois para analisar, tá? Tá tudo bem pra você?

**Sr.I5 (Entrevistado):** Tudo bem

**Entrevistadora** – sem problema, né? O sigilo é preservado, como tá lá no termo de consentimento, inclusive os nomes, durante a análise, como tá lá serão transformados em números ou em algum, em algum nome fictício, tá bom?

**Sr.I5 (Entrevistado):** tá...legal

**Entrevistadora** –Tá legal

**Entrevistadora** –eu não tava gravando ainda quando perguntei pra você se você concorda em participar da pesquisa. Então eu vou perguntar de novo se você concorda em participar da pesquisa. Se tudo bem pra você. Você já leu o termo, não é? Você concorda em participar da pesquisa?

**Sr.I5 (Entrevistado):** Sim.

**Entrevistadora** –Ok, então tá bom. Então tá I5. É...I5, é...conforme você pode ver no termo né, e no breve resumo que a gente faz ali do projeto, o objetivo dessa pesquisa é buscar os significados e sentidos que vocês, médicos residentes, possuem sobre a saúde bucal do pacientes com transtorno mental severo, né? Então queria começar com uma pergunta bem geral pra você; queria saber como é que você acha que isso se dá; como que na sua opinião tem se dado essa questão, né, da saúde bucal dos pacientes com transtorno mental para vocês, médicos, em geral?

**Sr.I5 (Entrevistado):** Cê fala...uma dúvida: você fala enquanto a minha formação pro serviço ou a I5 enquanto médica...porque assim...eu já terminei a psiquiatria, tô fazendo a especialização em psiquiatria infantil e já trabalho como psiquiatra

**Entrevistadora** –Aham. A I5 em geral: Qual o sentido desse assunto pra você na sua prática clínica?

**Sr.I5 (Entrevistado):** Tá.Hum...eu tô na especialização de psiquiatria infantil e a gente se preocupa bastante, principalmente com as crianças que têm algum déficit intelectual ou um autismo de moderado a grave ou até mesmo assim...as crianças com menos comprometimento. Porque, né? Porque é importante des lá desde a infância, que a criança se acostume...se acostume com o “ir ao dentista se acostume com a cadeira, se acostume com os barulhinhos, certo?

**Entrevistadora** –Certo

**Sr.I5 (Entrevistado):** Então assim...a gente se preocupa em desde a infância orientar os pais, orientar a mãe, você já procurou o odontólogo? É importante fazer uma revisão, conhecer ter algum vínculo, pra quando não chegar na idade adulta, não ter problema e se tornar - idade adulta adolescência, né? – não se tornar...não ter problemas e se tornar mais complicado esse contato. Em geral (ênfase no geral), quando eu atendo adultos, isso foi passado, tá...não foi estimulado, não ouve a ...(inaudível). A gente reforça: cê já procurou? Pra não deixar pra procurar só quando tá em a dor de dente ou quando tem o sofrimento, sabe? (inaudível). Sei lá da saúde bucal.

**Entrevistadora** –Pelo que você me diz, a a você percebe então que em geral a sua...a sua orientação é a primeira que eles recebem; não tinham recebido antes...

**Sr.I5 (Entrevistado):** das crianças em geral sim; dos adultos, alguns já receberam, mas existe uma certa dificuldade, tanto de marcação, ou de encontrar um profissional que seja mais habili...que seja habilitado em atender alguém num estado grave. .então, existem barreiras. Ahnnn...posso dizer que não foi a primeira orientação, mas foi juitos não chegam a...dentista

**Entrevistadora** –desculpa...deu uma cortadinha...muitos...

**Sr.I5 (Entrevistado):**: muitos nunca chegaram a ir ao dentista.

**Entrevistadora** –entendi

**Sr.I5 (Entrevistado):** É, pelo jeito, por barreiras

**Entrevistadora** –entendi. E pra você como é que é isso, de dar a primeira orientação? O que isso causa para você?

**Sr.I5 (Entrevistado):** Ahhh....eu acho que faz parte da minha entre aspas orientação com médica, né...pq a gente tem que olhar para a saúde global do paciente...não é só uma medicação, não é só encaminhar para o psicólogo, não é só isso, não e´? Ele é uma pessoa ali, integralmente e acho que como psiquaitra a gente tem sim que orientar, orientar atividade física, orientar a higiene do sono, orientar ida ao odontólogo, nas mulheres questionar: fez pappanicolaou esse ano? Como que tá sua ida ao ginecologista? E mamografia? E etc...eu acho. Pq enquanto médica, (risos) faz parte do meu mètiée...

**Entrevistadora** –e aí, I5, você nota que é isso é uma coisa comum entre vocês médicos, ou é uma preocupação você nota mais presente em você?

**Sr.I5 (Entrevistado):** Ah...eu não vou generalizar, tá? Eu acho que alguns residentes se preocupam, ou alguns...a grande maioria dos chefes também...eles alertam a gente e eles orientam, mas eu acho que...ah...nem todos se preocupam...

**Entrevistadora** –Porque será? Na sua opinião, o que pode ser entrave para isso?

**Sr.I5 (Entrevistado):** São vários fatores, viu Luciane, assim...Aqui na residência é diferente, né...a gente tá em processo de aprendizagem, mas no trabalho mesmo, na vida real entre aspas, as vezes tem pouco tempo de consulta, ou então tem váriso outros afazeres, ou então passa batido, bom, o que não justifica, porque a gente poderia fazer em outras consultas, mas eu acredito que por um lance de uma sobrecarga, talvez, profissional que, que, as orientações vão se perdendo, entendeu? Não justifica, tá? (risinho), mas existe.

**Entrevistadora** –E, e, quando você consegue passar isso às mães das crianças, como você disse, nem sempre elas conseguem o acesso ao serviço, mas a receptividade dela à sua orientação, como é que é?

**Sr.I5 (Entrevistado):** É bem variável. Então tem mãe que diz não, eu não vou levar pq ele é muito agitado, ele vai quebrar tudo, tem que ser...tem que dar um remédio. Ou então tem mãe que fala: legal, bom, bom lembrar...Na minha experiência - bom não é tão grande - mas eu não vejo um padrão, tá? Varia bastante.

**Entrevistadora** –E essa questão que você colocou da...de que entre os colegas há dificuldade pra fazer isso, você acha que é em relação a tempo, tempo de consulta, como você falou e muitas outras questões pra serem resolvidas e tratadas também...é uma questão relacionada a rotina de vocês. É isso? Ou tem mais fatores que possam interferir nessa relação com o paciente ou com a saúde bucal do paciente?

**Sr.I5 (Entrevistado):** É...e eu acho também a nossa formação, de uma certa forma. É preciso estar sempre lembrando, relembrando, de dar a devida importância a isso, sabe? Sim, tem a questão do tempo...é..é...eu acredito que precisa de uma educação continuada e ter semprte um olhar para a saúde bucal, sabe?

**Entrevistadora** –Aham. Você tá, você atende mais crianças, né.. pelo que você falou você está se especializando em psiquiatria infantil. É isso?

**Sr.I5 (Entrevistado):** Isso

**Entrevistadora** –Ah interessante. Eu imagino que você tenha de fato uma vivência com saúde bucal grande, uma vez que as crianças têm troca de dentição e uma série de questões que se apresentam ali e que no adulto não...Você convive com o assunto até mais que os outros.

**Sr.I5 (Entrevistado):** É. A gente vai orientando os pais Assim:. Você já levou? É importante. Lá pros 2 anos, 3 anos. Leva seu filho....ahhh as crianças com autismo, né que têm uma certa inflexibilidade....é importantíssimo pra que isso não seja um temor. e não só pras crianças que têm transtorno mental, (risos) pra todas as crianças mesmo.

**Entrevistadora** –claro, claro,você atende crianças de quanto a quanto – geralmente é de que idade a que idade? Só por curiosidade ...

**Sr.I5 (Entrevistado):** Bom, a criança mais nova que eu atendi acho que tinha 2 anos.

**Entrevistadora** –Nossa!

**Sr.I5 (Entrevistado):** Ou até um ano e dez meses. É bebê, tá? A gente tem um ambulatório de bebê e criança pequena que vai de zero....bom, menor idade que tiver até 5 anos. Depois temos o ambulatório que vai dos 5 aos 12 (até adolescência) e depois o ambulatório de adolescentes.

**Entrevistadora** –entendi. Então é...é variável...você tem uma faixa etária bem ampla, até, né I5?

**Sr.I5 (Entrevistado):** É...na residência de psiquiatria geral a gente atende mais adultos

**Entrevistadora** –É...eu ia chegar.. .ia lhe perguntar isso: você atende adulto também?

**Sr.I5 (Entrevistado):** Na clínica eu atendo.

**Entrevistadora** –Ah, tá. E com adulto, o que que muda? Tem diferença na sua atenção à saúde bucal entre a criança e o adulto? Como é que é?

**Sr.I5 (Entrevistado):** Pra ser sincera, sim. Acho que a gente...(inaudível), na criança...

**Entrevistadora** –desculpa...é que deu uma cortadinha aqui no começo... Entre adulto...você falou e cortou...

**Sr.I5 (Entrevistado):** Sendo bem sincera, no adulto a gente esquece.

**Entrevistadora** –Ah tá. E porque que você acha que isso se dá, I5?

**Sr.I5 (Entrevistado):** Bom, no adulto com transtorno mental grave, é...é..acredito que seja por dificuldade mesmo, do comportamento, ahhh...talvez da estrutura do serviço que vai recebê-lo...Então, da mesma forma que os pais da criança dizem que tem que sedar, que dar um remédio, o responsável pelo adulto com esquizofrenia – transtorno bipolar nem tanto porque eles andam em fases, né, quando estão bem, eles estão ótimos pra ir pro dentista - mas pra quem tem esquizofrenia mais comprometido, as vezes você não consegue marcar por conta disso...Porque fala que tem que ir no profissional que atenda esse tipo de paciente. Sabe? Eu fico “p” da vida com isso, mas eu já ouvi isso.

**Entrevistadora** –essa fala vem do paciente, que diz que ele gostaria de ir no profissional que só atende paciente...ou do dentista que dizendo que não vai atende-lo pq tem que encaminhar para o colega que só atenda?

**Sr.I5 (Entrevistado):** É o relato do paciente que diz: “Ah eu não consegui marcar”. Pq o caso dele é o caso de quem tem esquizofrenia e o dentista não atende esquizofrenia. Sabe? Eu já ouvi. Infelizmente!

**Entrevistadora** –Sim. E isso tem um impacto em você? OU naÕ? Como é? Como é ouvir isso?

**Sr.I5 (Entrevistado):** Ah...(riso) eu falo: -Como assim? Quer dizer que você não tem dente? (risos). Cê tem que falar isso lá! É obrigação! Ahhh...eu sou um pouco...eu sou pouco reativa, tá? Mas...eu falo pra tentar. Eu acho que eu já cheguei a fazer cartinha...eu já cheguei fazer cartinha solicitando atendimento odontológico. Pra paciente com esquizofrenia. (entrevistada balança afirmativamente cabeça em sinal de indignação).

**Entrevistadora** –certo.

**Sr.I5 (Entrevistado):** eu não lembro...eu não lembro se deu certo...mas deve ter dado certo...Alguns conseguem, outros não...Não é tão óbvio, tá? No paciente que não tem o transtorno mental grave...sei lá...que é uma ansiedade, uma depressão, ...que ele acaba sendo mais funcional isso normalmente não é lembrado, tá, Luciane? Exatamente pela funcionalidade do paciente, é...mas no que tem transtorno mental grave, a gente encontra algumas barreiras, dentre essas: de organização dos serviços.

**Entrevistadora** –E,a...a.... você falou que às vezes até você manda a cartinha, né? Essa ..a a volta dessa comunicação, existe também, ou não? A contra referência; o dentista se comunicar com vocês, costuma acontecer, ou não?

**Sr.I5 (Entrevistado):** Nunca, nunca...Não. Comigo, não. Comigo, não Aconteceu um caso interessante durante a residência. Eu era R1, no primeiro ano. A gente tinha um paciente com uma demência grave. E ele comia tudo que via pela frente. O Raio X dele tinha pedra, nas fezes tinham folhas, tinha tudo; e aí a gente procurou o dentista, os dentes dele muito estragados assim...e tinha fístula, sabe?. A gente procurou atendimento aqui na Unicamp, ele tava internado. E foi súper legal o que eles fizeram por esse paciente. Foi muito legal. Mas, na Unicamp, né?

**Entrevistadora** –você acha que tem uma diferença? Porque será que tem essa diferença, você acha?

**Sr.I5 (Entrevistado):** Ahhh...não sei...Digamos assim; vamos lá...deixa eu pensar: Ahhh...existe...pontualmente falando existem profissionais e profissionais...as vezes eu acho...talvez uma cultura do serviço, talvez... ah..não, na talvez Unidade básica a gente não atenda isso, que nem isso: a paciente tem esquizofrenia e eu não tô apta a tender. É...tem um pouco de cada coisa: de cultura do serviço, da maneira do profissional trabalhar, das dificuldades estruturais daquela UBS, então...ahh..não atendo essa complexidade. Porque será que não atendem essa complexidade? Será que eles não recebem? Será que é a estrutura, será que é a capacitação dos profissionais? Assim.. tem que ver o outro lado que não está aceitando , sabe?. Talvez eles tenham os motivos deles... Mas quais, né?

**Entrevistadora** –Sim

**Sr.I5 (Entrevistado):** Assim ...não que eu esteja apoiando alguém falar:

“- não atendo você pq você tem esquizofrenia”,

Mas, assim, minimamente questionar:

“-O que significa ter esquizofrenia?”

“- Qual que... ahhh...como deveria sern... pra você atender?

O que você precisaria ter pra atender?”

“ - O que que tá faltando?”

Eu acho que é necessário haver um diálogo, sabe?

**Entrevistadora** –E esse diálogo não está acontecendo, na sua opinião?

**Sr.I5 (Entrevistado):** Não, aqui...não...em geral não acontece.

**Entrevistadora** –E isso poderia favorecer muito o cuidado do paciente, né?!

**Sr.I5 (Entrevistado):** Sim, sim, sim, sim, sim. Muito, e muito mesmo. Porque assim ... considerando, por exemplo: transtorno mental Grave – esquizofrenia – ah...as vezes o paciente agita e ... a gente não sabe dizer de onde tá vindo essa agitação e ele tá quebrando as coisas em casa, e mudou o comportamento... As vezes ele tá sentindo dor; dor no dente e ele não sabe que tá sentindo dor no dente, entende? (Risos)...Então isso deveria ser obrigatório, sabe? Uma visita ao dentista ... uma vez por ano...não sei qual são os protocolos de vocês... mas é ... essa vigilância mesmo, com esses pacientes, porque vai ver e tá com dor de dente. Nossa...tinha muito! Na enfermaria tinha muito! Ei acho que quase todos os pacientes, eles passavam com dentista, sabe? Pq a gente entende que isso é um fator que pode agitar. Pra descompensar....

**Entrevistadora** –E facilitaria muito o tratamento de vocês, pelo que você tá falando, obviamente lidar com essa agitação, resolvendo a origem ao invés de medicar, inclusive.

**Sr.I5 (Entrevistado):** É ... pq tratamento não é só um remédio...

**Entrevistadora** –Perfeito

**Sr.I5 (Entrevistado):** Bom eu, pelo menos acho acredito que não seja um remédio.... Remédio tem sua importância, Óbvio. Não tô tirando a importância da medicação. Mas a gente precisa ver globalmente o paciente... as questões clínicas, ele tem um coração..ele pode enfartar, pode ter colesterol alto, pode ser obeso, né? ! As nossas medicações engordam. Ah...são pacientes que têm prejuízo funcional...na funcionalidade; será que ele tá escovando os dentes? Será que ele sabe escovar os dentes?

**Entrevistadora** –Faz sentido, então, essa preocupação?

**Sr.I5 (Entrevistado):** Muito! Total sentido! Desde a infância, tá? (inaudível)

**Entrevistadora** –Muito bem, I5. Nossa, você acrescentou muito pra gente...inclusive com alguns exemplos bons...da dor de dente, da dor relacionada a boca interferindo...Iso é muito importante. Tem alguma coisa mais que você queria acrescentar que talvez eu não tenha perguntado e que você gostaria de acrescentar ao assunto?

**Sr.I5 (Entrevistado):** Ahhh...eu acho que a gente poderia...achei muito interessante o tema da pesquisa...é... e acho que a gente precisaria conversar mais tanto pra minha prática clínica, quanto pra vocês mesmos....assim, pra dúvidas dos dentistas. O que é que pega em atender um esquizofrênico? O que é que cê tem medo? O que é que acontece? Então uma troca que eu acho que seria muito válida nas 2 formações, tá? Na, na formação de psiquiatra, na formação de dentista ....e acho que que isso acrescentaria bastante para o paciente.

**Entrevistadora** –Você vislumbra, consegue imaginar uma forma de isso acontecer na prática? Como poderia ser?

**Sr.I5 (Entrevistado):** Ó: aqui na na na Unicamp tem o responsável pela Odontologia; então de repente, que tivesse...residência por exemplo? E que o residente da odontologia passaria numaa enfermaria de saúde mental...não precisaria ser todo dia, mas ahhh... um turno na semana ele vai e avalia os pacientes.... Ou passaria no Capes, por exemplo, que faz parte da rede. Então, ele, ele...e aí sim, existiria uma troca. Ãaa... na verdade seria...é... talvez assim... E a agente também poderia tirar nossas dúvidas: quando que a gente deve chamar? Qual a frequência da avaliação? o que que a gente tem que observar? Porque também fica meio...obscuro pra gente, sabe?! Eu acho é essa pauta de, de troca, né?! Mas eu acho que é estando mais inserido, sabe, nos serviços...desde a formação, claro.

**Entrevistadora** –Sim

**Sr.I5 (Entrevistado):** Não sei ...mas talvez na faculdade... não sei...no estágio prático

**Entrevistadora** –Você imagina o médico também – fazendo o caminho inverso – o médico participando um pouco do cenário odontológico para adquirir alguma prática, ou não?

**Sr.I5 (Entrevistado):** Também. Tem que ter, né?! Acho que tem que ter as duas vias. Eu tô pensando como que a gente iria, entende ?! Não sei se por formações, ou aulas ou orientações...pensando no caminho inverso, né?...mas, eu acho que precisa... acho que esse diálogo é muito necessário. A gente fez um encontro sobre autismo, há pouco tempo...acho que setembro e a gente teve uma palestra e não me lembro o nome de quem deu a palestra (Não sou muito boa de nome) Me lembro mais do rosto dela...Falando da saúde bucal do paciente com autismo, das dificuldades enfrentadas por vocês, dos desafios, das necessidades.... Porque eu sei que também existe, entende? ...Quando ele fala “ahh não atendo esquizofrênico”, eu sei que não é um atendimento óbvio. O autista não é um atendimento óbvio. Então...por isso a formação, sabe? A necessidade da formação e da desconstrução de cultura

**Entrevistadora** –Muito bom, R. Olha, foi super rica a nossa conversa, viu?! Nossa entrevista... muito boa mesmo....Te agradeço demais, não quero ficar tomando seu tempo. Ficaria aqui batendo papo com você, um tempão, mas é... não faz sentido...você tá no trabalho, né? Mas, acho que a gente tirou muitas coisas importantes daqui...

**Sr.I5 (Entrevistado):** O que eu puder contribuir...Colaborar até pra gente entender as dificuldades que impedem um melhor cuidado ao paciente.

**Entrevistadora** –Muito bom, I5. Muito obrigada, I5.

**Áudio: LU06**

**Entrevistadora** – como você pode ver no termo, nosso objetivo é esclarecer algumas questões que permeiam a atividade profissional de vocês no atendimento ao paciente com transtorno mental e, no caso dessa pesquisa, nós gostaríamos de saber, é, como é que você vê a questão da saúde bucal do paciente com transtorno mental, como é que isso se dá, a relação do médico psiquiatra com a saúde bucal desses pacientes?

**SR.I6 (ENTREVISTADO): :** É…, a gente reconhece que tem a importância, o problema é que às vezes a gente acaba se descuidando mesmo de perguntar para os nossos pacientes, assim, se eles estão tendo o cuidado, é, tanto a própria higiene feita pelo paciente, até mesmo a ida ao dentista, e as vezes isso acaba sendo um pouco descuidado assim, eu creio que pelo menos da minha parte, assim.

**Entrevistadora** –E certamente é um assunto que é, vocês consideram importante mas passa.

**SR.I6 (ENTREVISTADO): :** Sim, uhum

**Entrevistadora –:** Você acha que isso passa, por que, quais razões, o que dificulta pra vocês?

**SR.I6 (ENTREVISTADO): :** ah, acho que no meu entender do, tanto do clínico geral também, às vezes isso acaba passando também, tanto na formação médica. É…, muitas vezes até os próprios pacientes trazem essa preocupação pra gente em relação aos dentes, e às vezes a gente acaba se cuidando mais principalmente nos pacientes que acabam reduzindo o auto cuidado de, as vezes deixando de escovar os dentes, se descuidando de outras coisas também, da higiene pessoal. As vezes eu acho que seria mais por conta da formação, assim, não ter o hábito de, de questionar isso do nosso paciente.

**Entrevistadora –:** Entendi, e isso você acha que se dá em todas as outras áreas ou é especificamente com a saúde bucal, por uma questão de formação, de gap, de falha aí na formação?

**SR.I6 (ENTREVISTADO): :** De outras áreas, você diz, em outras especialidades?

**Entrevistadora –:** É, outras áreas da saúde

**SR.I6 (ENTREVISTADO): :** Ah, sim

**Entrevistadora –:** Outras questões que o paciente tenha que não sejam bucais e nem psiquiátricas

**SR.I6 (ENTREVISTADO): :** hum, algumas coisas você acaba, assim, é, pesquisando mais, questão de peso, é… atividade física, que às vezes por serem tão presentes, assim, às vezes até de efeitos colaterais das nossas medicações, você acaba sendo mais ativo de perguntar, mas as vezes, eu acho que a saúde bucal a gente acaba descuidando um pouco.

**Entrevistadora –:** E constatar isso, como é que é pra você? Você tá me dizendo que tem essas dificuldades, que é uma falha talvez na formação e chegam pacientes com problemas bucais graves pra você?

**SR.I6 (ENTREVISTADO): :** Chegam, uhum

**Entrevistadora –:** E aí, como é que é quando vocês recebem esses pacientes?

**SR.I6 (ENTREVISTADO): :** Às vezes até quando eles trazem essa demanda de querer assim arrumar os dentes, é… ou mesmo assim quando eles internam, ficam numa enfermaria, a gente acaba tendo um pouco mais de cuidado, assim nessas mesmo chamar a Odonto, é… ou mesmo a gente se preocupar em ver isso também, mas às vezes assim, ambulatorialmente acho que é uma coisa que passa mais, assim, fica mais descuidado de ser questionado pro paciente

**Entrevistadora –:** Entendi, e… e os problemas são a frequência, prevalência dos problemas nessa população é grande mesmo? Na prática vocês veem isso?

**SR.I6 (ENTREVISTADO): :** Aham, sim

**Entrevistadora –:** A literatura mostra pra gente que sim né, como você viu lá no termo, eu coloquei, a literatura mostra que a prevalência de cárie nessa população é maior que na população em geral né

**SR.I6 (ENTREVISTADO): :** Uhum

**Entrevistadora –:** Mas isso pra vocês é claro, aparece mesmo na prática né

**SR.I6 (ENTREVISTADO): :** sim, uhum

**Entrevistadora –:** E daí quando aparece, pelo que você está me dizendo, vocês tem que buscar algum meio né, de resolver isso

**SR.I6 (ENTREVISTADO): :** Aham, ou pelo menos encaminhar ou de uma certa forma, é… de uma interconsulta

**Entrevistadora –:** Entendi, e quando vocês encaminham, obtém algum retorno? Tem algum feedback do que aconteceu?

**SR.I6 (ENTREVISTADO): :** Mais pacientes da enfermaria sim, pelo menos a gente vê ou se realmente tem uma indicação de fazer alguma resolução internado ou se é mesmo o tratamento ambulatorial.

**Entrevistadora –:** Entendi. Em geral dá pra resolver né, quando é na enfermaria, o encaminhamento acaba sendo efetivo?

**SR.I6 (ENTREVISTADO): :** É, esse ano, exemplo, por conta um pouco da pandemia né, as nossas inter consultas com a odonto era mais casos de urgência, então a gente acabou tendo, é… grandes casos assim que a gente precisou, mas a maioria deles era tratamento ambulatorial

**Entrevistadora –:** E, quando necessita ter essa inter consulta com o dentista, é, como é que isso se dá? É tranquilo, enfim, como que isso se dá?

**SR.I6 (ENTREVISTADO): :** É, no caso da enfermaria em hospital, a gente tem uma equipe já da Odonto disponível no hospital, a gente pede a interconsulta, pede uma avaliação, eles vão até o paciente e eles dariam a conduta, isso... hospitalar

**Entrevistadora –:** Mas a comunicação entre vocês, como é que ela se dá? Entre médico e dentista

**SR.I6 (ENTREVISTADO): :** Assim, eles são solícitos assim, quando a gente pede eles são solícitos, é… uma relação boa assim, a gente não teve grandes problemas, eu acho

**Entrevistadora –:** E eles intervêm quando são chamados? É isso né

**SR.I6 (ENTREVISTADO): :** Isso, é, eles fazem a avaliação e aí se tem alguma intervenção eles dizem, senão é só o tratamento ambulatorial, sem condutas pela equipe

**Entrevistadora –:** Entendi, da mesma forma vocês também fazem isso com outros profissionais né

**SR.I6 (ENTREVISTADO): :** Outras especialidades, isso, uhum

**Entrevistadora –:** E você acha que tem diferença nessa relação, de vocês com outras especialidades porque também são médicos e de vocês com dentista, ou nao? É...

**SR.I6 (ENTREVISTADO): :** Ah, eu particularmente acho que não, assim, vejo da mesma forma, é, não vejo diferença

**Entrevistadora –:** Tem, é, esse possível gap na formação, que você estava citando, é, isso você nota em outras áreas ou é especificamente na saúde bucal?

**SR.I6 (ENTREVISTADO): :** Humm, eu, assim, acho que considerando a saúde bucal aquilo que a gente tá falando, é o que eu vejo, assim na formação como clínico geral, assim que, até mesmo clínico geral que vê todas as outras especialidades, assim, perguntar da saúde bucal é uma coisa mais, que às vezes passa despercebido mesmo, não só pelo psiquiatra, essa é minha… ideia.

**Entrevistadora –:** Entendi, e já aconteceu com você alguma vez de estar diante de algum problema, é , saúde bucal que requer algum encaminhamento e você não ter com quem contar, a não ser na enfermaria quando você tem a equipe, em outras situações que você não tem como resolver ?

**SR.I6 (ENTREVISTADO): :** Isso nunca aconteceu, mas também porque eu não tive grandes experiências fora do hospital, assim, as principais, é hospitalar, no primeiro ano. Até em centro de saúde, assim, a gente acabou não tendo muito essa experiência esse ano, então eu nunca cheguei a ter, a acontecer isso

**Entrevistadora –:** Entendi, e você quer contar alguma experiência que você tenha tido com saúde bucal ou com...

**SR.I6 (ENTREVISTADO): :** Deixa eu pensar…

**Entrevistadora –:** Tem alguma que você possa relatar pra gente, algum caso aí que tenha te chamado atenção

**SR.I6 (ENTREVISTADO): :** Olha, específico mesmo eu… eu lembro muito dos casos dos outros residentes que tiveram pacientes,

**Entrevistadora –:** Tudo bem, tá tranquilo, pode ser

**SR.I6 (ENTREVISTADO): :** Mas tem um caso que… que... eu tive que chamar a odonto, mas a minha experiência, mais assim, que eu vejo, pacientes que reduzem o autocuidado, assim, que não tem uma higiene pessoal mínima, e aí quando a gente vai ver, realmente a gente acaba não perguntando mesmo se ele tem escovado os dentes, como que os dentes dele estão, se ele tem ido ao dentista com uma certa regularidade, eu acho que é um pouco mais dessa experiência, assim, é… assim, até experiência assim como médica, fora da questão psiquiátrica, assim, as vezes é perguntar mesmo pro paciente que veio com queixa de febre, alguma, se tem alguma alteração bucal, se os dentes dele tão em bom estado, isso é meio, é um pouco difícil também a gente acabar perguntando assim na clínica médica também. As vezes considera as vezes que é uma infecção de garganta, alguma coisa do tipo e a gente não pergunta do dente.

**Entrevistadora –:** Entendo, entendo, por que você acha que isso acontece?

**SR.I6 (ENTREVISTADO): :** Eu acho que é um pouco da formação, assim

**Entrevistadora –:** Volta nessa parte aí, entendi

**SR.I6 (ENTREVISTADO): :** É, eu acho que é na questão da formação, assim de, querendo ou não a nossa formação é saber perguntar, é saber avaliar, saber investigar, e se isso não faz parte do nosso questionar, nosso aval de questões, assim, a gente acaba não criando um padrão de que tem que ter, perguntar sobre a saúde bucal, acho que esse é o meu ponto de vista, assim

**Entrevistadora –:** E você teria alguma sugestão pra tentar resolver isso, suponhamos que você pudesse fazer algo nesse sentido pra… pra vencer essa barreira, o que você sugeriria?

**SR.I6 (ENTREVISTADO): :** Acho que assim, tanto da questão da formação que já foi citada, mas as vezes eu vejo que os meus professores já perguntam um pouco sobre a saúde bucal, então acho que vem um pouco também da experiência de você saber que é necessário investigar isso, saber se o paciente tá tendo esse cuidado, se o paciente tá indo atrás disso, é… eu vejo mais assim dos meus professores, eles perguntam, a questão do dente, e às vezes a gente não, por não estar tão habituado.

**Entrevistadora –:** O médico que já tá formado, já teve essa “falha”, enfim, essa, já decidiu a formação e já tá formado, o que você sugere que aconteça pra que isso possa ser, por exemplo, no seu caso, tem uma experiência, pelo que você tá me dizendo, menor ainda né

**SR.I6 (ENTREVISTADO): :** Uhum

**Entrevistadora –:** Então você não tem toda a experiência, não tem a formação também, mas, para resolver esse problema, o que você sugere?

**SR.I6 (ENTREVISTADO): :** Olha, difícil haha, acho que é um pouco parar pra pensar nisso mesmo né, acho que humm, é ir se acrescentando, a formação pós, pós formado, é, de ter essa avaliação, isso que é importante

**Entrevistadora –:** Você tá me falando de uma responsabilidade de um médico né, e carregando bastante isso pra você, pra vocês médicos mas e o paciente, como é que é nessa história? Suponhamos que você tenha como orientá-lo, ou como encaminhá-lo, é, vai ser … pra ele fazer o que você tá sugerindo ou encaminhando, ou vai ter dificuldades do outro lado também, o que você acha?

**SR.I6 (ENTREVISTADO): :** Sim, eu acho que têm dificuldade sim, mas às vezes quando o paciente vem às vezes um pouco com essa demanda, que assim é uma coisa que incomoda, da questão da autoestima, questão do dente, e acho que às vezes isso eles acabam indo um pouco mais atrás também, quando é alguma coisa que incomoda eles, eu acho assim, eles trazem já essa demanda, eu acho que isso fica um pouco deles irem atrás também. Mas quando o paciente não vem muito com essa demanda, já tem um pouco dessa dificuldade de, de ter o compromisso com o autocuidado, é bem mais difícil mesmo, assim, bem difícil

**Entrevistadora –:** Entendo, entendo, aí soma-se todos os fatores contra né

**SR.I6 (ENTREVISTADO): :** É, uhum

**Entrevistadora –:** É, isso que você tá me dizendo, legal, muito bom. É, basicamente era isso, SR.I6 (ENTREVISTADO): , eu te falei que é rápido né, na verdade a gente precisava, a gente tá buscando entender as percepções de vocês sobre o assunto, em primeiro lugar, pra depois trabalhar isso e poder devolver pra vocês em forma de, é, de sugestões e manejo do assunto, enfim, é, devolver o que tá acontecendo de uma forma positiva que possa melhorar essa interface aí né, entre vocês, essa relação entre vocês e paciente no que diz respeito a essa interface saúde bucal, né. Se você quiser acrescentar mais alguma coisa.

**SR.I6 (ENTREVISTADO): :** Ah, acho que é só isso mesmo, assim, mas a gente reconhece que é importante, é uma coisa que passa muito batido assim, pra gente.

**Áudio: LU07**

**Entrevistadora –** Como você pôde ver, o objetivo da nossa pesquisa é compreender melhor a saúde bucal no contexto de vocês psiquiatras né, como é que vocês psiquiatras enxergam essa questão da saúde bucal dos seus pacientes com transtorno mental, né. Que significado tem isso pra vocês, como é que você acha que isso se dá com os médicos psiquiatras em geral?

**SR.I7 (ENTREVISTADO):** Olha, eu particularmente tive algumas experiências relacionadas a isso que marcaram a minha formação, por exemplo, durante a minha residência tive um paciente que tinha um quadro de é… retardo mental grave desde a infância, é... não verbal, acamado. Ele foi internado porque todos os dentes dele estavam quebrados, ele não tinha nenhuma condição, ele gritava o dia inteiro de dor, demorou muito pra isso ser percebido, nesses pacientes. Então, foram, eu tive algumas experiências que então a equipe da bucomaxilo cuidou desses pacientes, são pacientes que a gente teve que tirar todos os dentes né, que aquela, uma experiência bem… bem importante… bem, foi bem demorado assim na enfermaria, até depois pra adaptar a questão depois, alimentação e é uma questão muito importante que eu vejo que é muito pouco abordada, muito pouco abordada. O, no sus, o sus no geral tem, tem pouco, é… consegue fazer pouco pela população em relação a saúde bucal e eu vejo que em pacientes, como eu sou psiquiatra infantil, o autista, alguns... alguns pacientes teriam que ter uma atenção mais próxima né, mais frequente em relação a saúde bucal e a gente sabe que isso não acontece.

**Entrevistadora –** Uhum, e por que você acha que é assim? Por que você acha que é dessa forma, como você imagina que pudesse ser diferente?

**SR.I7 (ENTREVISTADO):** Olha, é, eu acho que é, bom… é uma questão que eu acho que depende de um trabalho de equipe multidisciplinar né, eu acho que envolve um déficit da atenção básica que é antigo e que envolve toda uma equipe né, não falta só dentista, falta também, é… terapeuta ocupacional, fisioterapeutas na rede, assim, falta, a nossa rede é muito escassa, eu acho que poderia beneficiar se realmente funcionasse, se é, poderia ser, digamos, mais efetivos se a teoria realmente funcionasse, porque a gente vê que a teoria não funciona na prática, assim, no serviço único de saúde, em relação ao atendimento à população, eu acho que de uma maneira geral a saúde bucal é super importante, mas, por exemplo, eu tenho amigo que é dentista na Inglaterra e fala que brasileiro tem uma relativa, dá uma relativa importância pra saúde bucal até e, por exemplo, lá na Inglaterra ele fala que a situação é muito mais, muito pior de saúde bucal dos ingleses, assim, e que isso envolve toda uma questão cultural também.

**Entrevistadora –** E o médico psiquiatra, como que ele tem atuado nesse sentido, da saúde bucal dos seus pacientes? Vocês falam sobre isso com o paciente, como é que é?

**SR.I7 (ENTREVISTADO):** A gente fala, na psiquiatria infantil a gente fala, a gente acompanha esse pacientes e avalia se, e orienta os pais a procurarem desde cedo o dentista, acostumar a criança a ir ao dentista, ao toque na boca, essa questão toda né, que a gente sabe que quanto mais tarde inicia essa procura, é… ou mesmo esses cuidados de higiene, a gente orienta muito também, que é pra escovar os dentes mesmo se fizer birra, sempre vai ser difícil sim, não vai ser fácil né porque, é… a gente faz, na psiquiatria infantil a gente faz. Quando eu fazia residência geral, psiquiatria geral, isso era muito pouco abordado, no consultório, que a gente tivesse tipo essa cobrança, por exemplo, com o paciente adulto a gente não tem essa preocupação mas na infantil a gente tem mais, a gente tem, tem tido mais assim, o nosso acompanhamento envolve essa pergunta, tanto todas as terapias, tanto a terapia ocupacional, a fono, tudo a gente pede pro dentista também.

**Entrevistadora –** Por que você acha que no adulto é menos?

**SR.I7 (ENTREVISTADO):** Pois é, porque eu acho que no adulto envolve mais uma questão cultural de higiene mesmo, e uma coisa que eu acho que na psiquiatria infantil envolve a questão sensorial e comportamental mais importante, assim, eu acho né, nessa primeira infância, assim, nessa formação de hábito, eu acredito.

**Entrevistadora –** Você é R4 já né?

**SR.I7 (ENTREVISTADO):** Isso, psiquiatria infantil

**Entrevistadora –** Legal, você atende criancinha pequenininha até, de quanto em quanto mais ou menos?

**SR.I7 (ENTREVISTADO):** A gente tem um ambulatório na sexta feira que vai de 0 a 5 anos, mas vai criança em média de 2 a 5 anos. E aí tem um ambulatório na quinta de manhã que é de 5 a 10 anos e na quarta a tarde são os adolescentes

**Entrevistadora –** A tá, e você atende adolescente também?

**SR.I7 (ENTREVISTADO):** Também, uhum.

**Entrevistadora –** E com eles, como é que é? É complexo isso ou não, como é que é?

**SR.I7 (ENTREVISTADO):** Com eles a gente aborda quando tem o autismo, quando tem é… essas questões assim, quando é não verbal, quando precisa de uma atenção maior, a gente aborda a questão dos dentes também. Mas quando é um adolescente, por exemplo, que vai por ansiedade, outras coisas, aí eu acho que o psiquiatra atua menos na questão do dentista, assim.

**Entrevistadora –** Você já chegou a tocar no assunto nesses casos?

**SR.I7 (ENTREVISTADO):** Depende muito, por exemplo, eu já tive pacientes que vieram me procurar por coisas que aconteceram no dentista, por exemplo, depende...

**Entrevistadora –** A, é?

**SR.I7 (ENTREVISTADO):** É, eu tive uma paciente da minha cidade que ela foi num dentista desses populares e ele arrancou grande parte dos dentes dela, falando que ia fazer implante e ela teve problemas psiquiátricos seríssimos com tudo isso, então era uma temática importante né, aí são casos e casos. Eu acho que o… pra esses outros pacientes, é, a questão da saúde bucal ela não é tão enfática quanto pra esses que a gente sabe que não tem tanta autonomia de auto cuidado. A gente se preocupa mais nesses que… que exigem mais, porque às vezes até os pais não percebem quanto as questões do dente é importante pra aquela criança, etc.

**Entrevistadora –** Pelo que eu to entendendo, muitas vezes você acaba sendo o primeiro alerta, né?

**SR.I7 (ENTREVISTADO):** Sim, às vezes sim, se o pediatra não olhar a gente acaba tendo que descobrir. Quando tem muita agitação os nossos chefes falam né, quando mudou muito o comportamento etc você tem que olhar também os dentes, a saúde bucal.

**Entrevistadora –** Hum, interessante isso, é, passou a ser um alerta pra vocês, de repente uma informação, tem que olhar a saúde bucal também né

**SR.I7 (ENTREVISTADO):** É, os pacientes, principalmente os autistas, a gente olha sim.

**Entrevistadora –** Hum, muito bom. E na sua opinião, poderia ser diferente, assim, há alguma sugestão que você poderia fazer no sentido de aprimorar, melhorar, é, essa multidisciplinaridade? Você falou de ter mais, ter mais profissionais na rede, né?

**SR.I7 (ENTREVISTADO):** É

**Entrevistadora –** Só isso ou teria mais alguma sugestão?

**SR.I7 (ENTREVISTADO):** Eu acho que seria importante que tivesse um intercâmbio de tudo isso né, dessa funcionalidade, uma troca de tudo isso né, não só cada um indo no local, como teoricamente deveria funcionar, mas a gente sabe que a teoria não funciona. É porque a demanda populacional é muito grande, o ideal era que, por exemplo, assim, num paciente da psiquiatria infantil tivesse uma rede multidisciplinar que se comunicasse e fizesse todo o, uma abrangência integral desse paciente né, de forma integrada, assim, esses profissionais pudessem, que a fono pudesse se comunicar com o dentista, com a T.O., com todo mundo. Por exemplo, algumas estereotipias, algumas coisas. Eu tive uma paciente autista que teve lesão em córnea por uma estereotipia, sabe, são coisas que, por exemplo, você tem uma equipe que conversa, com uma terapia ocupacional, tudo isso você consegue integrar melhor o cuidado, e a gente vê que isso não acontece. Teoricamente isso deveria acontecer, em caps, em algumas redes, mas na prática não acontece porque é muita, muita, muito paciente pra pouca equipe.

**Entrevistadora –** Uhum. Você comentou do seu primeiro caso né, o primeiro caso que você exemplificou aí, o paciente acabou perdendo muitos dentes, como é que foi pra você ver isso, o que isso impactou?

**SR.I7 (ENTREVISTADO):** Impactou pra mim?

**Entrevistadora –** Como você se sentiu?

**SR.I7 (ENTREVISTADO):** É… o que eu senti foi muita angústia, na verdade, até que aqueles dentes foram retirados, porque eu pensei em quanto esse paciente sofreu, por quanto tempo sem que… que isso fosse feito nada sabe, acho que a minha maior angústia foi por tudo o que ele passou, por quanto tempo, ele tinha dois pais muito idosos, pais que tinham oitenta e poucos anos e ele ficava num quarto isolado e gritando de dor, há muito tempo, então isso foi o que mais me impressionou sabe, a situação que estava aqueles dentes, até a equipe de buco ficou também chocada com tudo, a situação que estavam os dentes, realmente não tinha como, vários quebrados, até verticalmente, assim, sabe? Então, foi uma coisa que ficou, depois que ele tirou os dentes, assim, a questão de impacto, de...estética, algo assim, acho que… ele ficou tão melhor, o semblante dele, tudo, que isso não, assim, não...não teve impacto, assim, foi como se fosse um alívio, saber que realmente você tava melhorando a qualidade de vida daquela pessoa, sabe?

**Entrevistadora –** E o outro caso que você comentou da paciente que foi procurar o dentista, que tava perdendo vários dentes

**SR.I7 (ENTREVISTADO):** Aham, é, essa eu não tive mais muita continuidade. Essa vai ser um grande problema.

**Entrevistadora –** Entendi, a orientação pra uma criança né, no seu caso que é psiquiatra infantil, faz uma primeira orientação e a mãe acaba adquirindo esse hábito né , da saúde bucal, de levar ao dentista etc, imagino que seja o contrário, seja muito gratificante isso né, pra você

**SR.I7 (ENTREVISTADO):** Sim, com certeza

**Entrevistadora –** A orientação de vocês, geralmente, é no sentido de levar ao dentista, é isso? Vocês fazem mais orientações, tem mais alguma orientação que vocês costumam dar sobre saúde bucal?

**SR.I7 (ENTREVISTADO):** Ah, além de perguntar se está fazendo a escovação adequada, a gente pergunta isso, se tá conseguindo fazer, como, como...como está fazendo né, e se não a gente encaminha mesmo, encaminha, pra outros tipos de orientações, assim, mais específicas a gente acaba encaminhando. A gente fala sobre o hábito de escovar os dentes, três vezes ao dia, pelo menos, com os pais, mas mais detalhes em relação a isso a gente não, geralmente não costuma dar.

**Entrevistadora –** E os pais recebem bem essa orientação ou eles sempre encontram barreiras do tipo “ah mas é difícil”, “ah mas não sei o que…”?

**SR.I7 (ENTREVISTADO):** Muitas, muitas barreiras, muitas barreiras, de que não conseguem, muitas, muitas. Muitas a gente vê que desiste, assim, a gente desconfia bem se é realmente feito. Muito de briga, briga, de ser uma briga, de constante briga.

**Entrevistadora –** Puxa, é um desafio né

**SR.I7 (ENTREVISTADO):** É um desafio, mas a gente vê pra todas as crianças são né, geralmente, na infância, não é fácil fazer criança escovar o dente né, não é uma coisa simples assim, então...

**Entrevistadora –** É, interessante, se, em relação a essas informações que vocês tem que dar, isso é tranquilo pra você, você sente alguma dificuldade do ponto de vista técnico mesmo, ter alguma dificuldade pra vocês ou é tranquilo? Eu digo, as orientações técnicas sobre saúde bucal para os pacientes.

**SR.I7 (ENTREVISTADO):** Ah, é, a gente recebe, a gente tem poucas assim, seria até interessante a gente ter, saber um pouco mais pra orientar, eu acho.

**Entrevistadora –** Uhum, tá.

**SR.I7 (ENTREVISTADO):** Seria interessante a gente receber às vezes, algumas coisas importantes que seriam legais a gente indicar né, porque nunca foi realmente, digamos, explanado isso pra nós assim.

**Entrevistadora –** É, a atuação de dentista, e do Cecom ou de algum outro setor da Unicamp com vocês, é… em algum, é, ou no ambulatório ou na enfermaria, tem alguma? Porque que eu saiba, regularmente não, se forem chamados por vocês eles vão, já teve algum caso assim?

**SR.I7 (ENTREVISTADO):** Ainda não, é, mas é porque como eu sou R4 é diferente isso, às vezes na enfermaria já teve né.

**Entrevistadora –** Entendi

**SR.I7 (ENTREVISTADO):** Mas da gente ainda não, e porque teve a pandemia também então, não teve muito, esse ano foi um pouco atípico né

**Entrevistadora –** É, de fato foi, até a nossa entrevista ficou assim meio diferente. Mas tá bom, nossa, você me ajudou demais porque deu segmentos importantes pra fazer a gente pensar, viu

**SR.I7 (ENTREVISTADO):** Ah, que bom.

**Entrevistadora –** Tem mais alguma coisa que você gostaria de acrescentar sobre isso, algo que eu não tenha perguntado sobre saúde bucal, e que de repente possa ser uma questão pra você?

**SR.I7 (ENTREVISTADO):** Não, o que eu acho que seria legal é se a gente pudesse realmente receber orientações de coisas importantes, pra passar para os pacientes e quem sabe ter até algum protocolo, alguma coisa, bem estabelecida né, de algo que seria legal pra gente indicar, um tempo, de tempos em tempos, quanto em quanto tempo ir ao dentista com tal idade, com tal diagnóstico, essas orientações básicas de higiene, algo mais profissional, bem elaborado seria legal a gente ter, seria muito bom.

**Entrevistadora –** Ótimo, vamos pensar né nas propostas pra melhorar

**SR.I7 (ENTREVISTADO):** Sim!

**Entrevistadora –** Muito bom I7, muito bom, eu te agradeço muito, viu, pela disposição em me ajudar, ajudar a nossa pesquisa, a ideia é devolver isso pra vocês de formas outras que possam colaborar aí né com vocês, tá bom?

**SR.I7 (ENTREVISTADO):** Legal!
